# Supplementary material for: MANF stimulates autophagy and restores mitochondrial homeostasis to treat autosomal dominant tubulointerstitial kidney disease in mice
Source: Nat Commun. 2023 Oct 14;14:6493. doi: 10.1038/s41467-023-42154-0 (PMC10576802; doi:10.1038/s41467-023-42154-0)

Supplementary Materials for

MANF stimulates autophagy and restores mitochondrial homeostasis to treat autosomal dominant tubulointerstitial kidney disease

Yeawon Kim,[1†] Chuang Li,[1†] et al.

[†] These authors contributed equally

[*]Corresponding author:

Ying Maggie Chen, MD., Ph.D., FASN
Washington University School of Medicine
Division of Nephrology, Campus Box 8126
660 S. Euclid Ave.
St. Louis, MO 63110
Tel: 314-362-4629
Email: ychen32@wustl.edu

Supplementary Table 1. List of primary antibodies

Antibodies	Source	Identifier	Clone #	Dilution
mouse monoclonal anti-β-actin-Peroxidase	Sigma	Cat# A3854	AC-15	1:20,000
rabbit monoclonal anti-AMPKα	Cell Signaling	Cat# 5832	D63G4	1:1,000
rabbit monoclonal anti-Phospho AMPKα (T172)	Cell Signaling	Cat# 50081	D4D6D	1:1,000
rabbit polyclonal anti-ATF4	Santa Cruz	Cat# sc-200		1:1,000
mouse monoclonal anti-ATF6	Novus	Cat# NBP1-40256	70B1413.1	1:1,000
rabbit polyclonal anti-BIP	Proteintech	Cat# 11587-1-AP		1:1,000 (WB), 1:50 (IF)
rabbit polyclonal anti-BNIP3	Cell Signaling	Cat# 3769		1:1,000
rabbit monoclonal anti-BNIP3L	Cell Signaling	Cat# 12396	D4R4B	1:1,000
rabbit polyclonal anti-Calnexin	Santa Cruz	Cat# sc-11397		1:50 (IF)
rabbit polyclonal anti-Caspase 3	Cell Signaling	Cat# 9662		1:1,000
mouse monoclonal anti-Caspase 9	Cell Signaling	Cat# 9508	C9	1:1,000
rabbit monoclonal anti-cleaved caspase 3	Cell Signaling	Cat# 9664	5A1E	1:1,000
rabbit polyclonal anti-Collagen I	Abcam	Cat# 34710		1:50 (IF)
rabbit polyclonal anti-COX IV	Cell Signaling	Cat# 4844		1:1,000
goat polyclonal anti-CRELD2	R&D Systems	Cat# AF3686		1:1,000
rat monoclonal anti-F4/80	Invitrogen	Cat#14-4801-82	BM8	1:50 (IF)
rabbit polyclonal anti-Fibronectin	Abcam	Cat# ab2413		1:1,000 (WB), 1:50 (IF)
rabbit polyclonal anti-FIS1	GeneTex	Cat# GTX111010		1:1,000
rabbit monoclonal anti-Foxo3a	Cell Signaling	Cat# 12829	D19A7	1:1,000
rabbit monoclonal anti-FUNDC1	Cell Signaling	Cat# 49240	E2F4T	1:1,000
rabbit monoclonal anti-GAPDH	Cell Signaling	Cat# 5174	D16H11	1:5,000
mouse anti-GFP	Roche	Cat# 11814460001	7.1 & 13.1	1:1,000
rabbit monoclonal anti-IRF3	Cell Signaling	Cat# 4302	D83B9	1:1,000
rabbit monoclonal anti-phospho-IRF3 (S396)	Cell Signaling	Cat# 29047	D6O1M	1:500
rabbit polyclonal anti-Laminin	Abcam	Cat# ab11575		1:2,000
rabbit polyclonal anti-LC3A/B	Cell Signaling	Cat# 4108		1:1,000
rabbit polyclonal anti-MANF	Abnova	Cat# PAB13301		1:1,000 (WB), 1:100 (IF)
goat polyclonal anti-MANF	R&D	Cat# 3748		1:50 (IF)
rabbit monoclonal anti-Mitofusin2	Cell Signaling	Cat# 9482	D2D10	1:1,000
rabbit polyclonal anti-Phospho mTOR (S2481)	Cell Signaling	Cat# 2974		1:1,000
rabbit polyclonal anti-NF-kB p65	Abcam	Cat# ab16502		1:1,000
rabbit monoclonal anti-phospho-NF-kB p65 (S536)	Cell Signaling	Cat# 3033	93H1	1:500
goat polyclonal anti-NGAL	R&D Systems	Cat# AF1857		1:1,000
rabbit polyclonal anti-NKCC2	Alpha Diagnostic	Cat# NKCC21-A		1:50 (IF)
OXPHOS antibody cocktail	Abcam	Cat# ab110413		1:2,000
rabbit polyclonal anti-p62/SQSTM1	Cell Signaling	Cat# 5114		1:1,000
mouse monoclonal anti-Parkin	Abcam	Cat# ab77924	PRK8	1:1,000
mouse monoclonal anti-PGC1a	Proteintech	Cat# 66369-1-AP	1C1B2	1:1,000
rabbit polyclonal anti-PINK1	Abcam	Cat# ab23707		1:1,000
mouse monoclonal anti-Smooth muscle actin	Sigma	Cat# A2547	1A4	1:1,000 (WB), 1:50 (IF)
rabbit anti-mouse monoclonal anti-STING	Cell Signaling	Cat# 50494	D1V5L	1:1,000
rabbit anti-human monoclonal anti-STING	Cell Signaling	Cat# 13647	D2P2F	1:1,000
rabbit monoclonal anti-TBK1	Cell Signaling	Cat# 3504	D1B4	1:1,000
rabbit monoclonal anti-phospho-TBK1 (S172)	Cell Signaling	Cat# 5483	D52C2	1:500
rabbit anti-TFAM	Sigma	Cat# SAB1401383		1:1,000
rat monoclonal anti-Uromodulin	R&D Systems	Cat# MAB5175	774056	1:1,000 (WB), 1:50 (IF)
rabbit anti-Uromodulin	Alfa Aesar	Cat# J65429		1:1,000
sheep polyclonal anti-Uromodulin, Biotin	R&D Systems	Cat# BAF5175		4 ug/mouse (TAL isolation)
mouse monoclonal anti-human Uromodulin	RayBiotech	Cat# 119-13298	10.32	1:1,000 (WB), 1:50 (IF)
rabbit polyclonal anti-XBP1s	BioLegend	Cat# 619502		1:1,000

Supplementary Table 2. Q-PCR primers for kidneys and TALs

Gene name	Forward primer	Reverse primer
18S rRNA	GTAACCCGTTGAACCCCATT	CCATCCAATCGGTAGTAGCG
Acta2	AGCCATCTTTCATTGGGATGGA	TACCCCCTGACAGGACGTTG
Actb	CAGCCTTCCTTCTTGGGTAT	GGTCTTTACGGATGTCAACG
Atg5	AGCCAGGTGATGATTCACGG	GGCTGGGGGACAATGCTAA
Atg7	GTTCGCCCCCTTTAATAGTGC	TGAACTCCAACGTCAAGCGG
Becn1	TGAAATCAATGCTGCCTGGG	CCAGAACAGTATAACGGCAACTCC
Bnip3	TCCTGGGTAGAACTGCACTTC	GCTGGGCATCCAACAGTATTT
Ccl2	TTAAAAACCTGGATCGGAACCAA	GCATTAGCTTCAGATTTACGGGT
Col1a1	AGACATGTTCAGCTTTGTGGAC	GCAGCTGACTTCAGGGATG
Fn1	CCAGAAACAGATGCAACGAT	GCAGACACACTGAAGCAGGT
Foxo3	CTGGGGGAACCTGTCCTATG	TCATTCTGAACGCGCATGAAG
Gabarapl1	GGACCACCCCTTCGAGTATC	CCTCTTATCCAGATCAGGGACC
Gapdh	TGTAGACCATGTAGTTGAGGTCA	AGGTCGGTGTGAACGGATTTG
Icam1	AGCACCTCCCCACCTACTTT	AGCTTGCACGACCCTTCTAA
IL1b	GCACTACAGGCTCCGAGATGAAC	TTGTCGTTGCTTGGTTCTCCTTGT
IL6	TAGTCCTTCCTACCCCAATTTCC	TTGGTCCTTAGCCACTCCTTC
Manf	CCTCAAAGACAGAGATGTCACA	TAGTAGCACAACCGATTCTC
Map1lc3b	TTATAGAGCGATACAAGGGGGAG	TTATAGAGCGATACAAGGGGGAG
mt-Co1	GCCCCAGATATAGCATTCCC	GTTCATCCTGTTCCTGCTCC
mt-Cytb	AGTAGACAAAGCCACCTTGA	CCGCGATAATAAATGGTAAG
mt-Nd4	GCCTCACATCATCACTCCTATT	GGCTATAAGTGGGAAGACCATT
mt-Rnr2	GTTACCCTAGGGATAACAGCGC	GATCCAACATCGAGGTCGTAAACC
Pink1	CACACTGTTCCTCGTTATGAAGA	CTTGAGATCCCGATGGGCAAT
Sqstm1	AGGATGGGGACTTGGTTGC	TCACAGATCACATTGGGGTGC
Tfam	GAGCAGCTAACTCCAAGTCAG	GAGCCGAATCATCCTTTGCCT
Tgfb1	TGGAGCAACATGTGGAACTC	CAGCAGCCGGTTACCAAG
Tnf	GGAACTGGCAGAAGAGGCACTC	GCAGGAATGAGAAGAGGCTGAGAC
Ulk1	AAGTTCGAGTTCTCTCGCAAG	CGATGTTTTCGTGCTTTAGTTCC

Supplementary Table 3. Q-PCR primers for stably transduced HEK 293 cells

Gene name	Forward primer	Reverse primer
18S rRNA	CTCAACACGGGAAACCTCAC	CGCTCCACCAACTAAGAACG
CCL2	CAGCCAGATGCAATCAATGCC	TGGAATCCTGAACCCACTTCT
GAPDH	GTCTCCTCTGACTTCAACAGCG	ACCACCCTGTTGCTGTAGCCAA
ICAM1	ATGCCCAGACATCTGTGTCC	GGGGTCTCTATGCCCAACAA
IL1B	AGCTACGAATCTCCGACCAC	CGTTATCCCATGTGTCGAAGAA
IL6	ACTCACCTCTTCAGAACGAATTG	CCATCTTTGGAAGGTTCAGGTTG
MT-CO1	GAGCCTCCGTAGACCTAACC	TGAGGTTGCGGTCTGTTAGT
SQSTM1/p62	GCACCCCAATGTGATCTGC	CGCTACACAAGTCGTAGTCTGG
TNFA	CCTCTCTCTAATCAGCCCTCTG	GAGGACCTGGGAGTAGATGAG
UMOD	GCGTACTGCACAGACCCCAGC	GTCATTGAAGCCCGAGCACCG

Supplementary Table 4. PCR primers for genotyping

Allele	Forward primer	Reverse primer
P8TA	CCATGTCTAGACTGGACAAGA	CTCCAGGCCACATATGATTAG
Umod $^{DEL/+}$	TTTACAGGGGATGGTTGGTACTGTGAG TGC	TCGAGGGATGAGAGCCATTGAGCCACAT GG
TET-MANF	ATCCACGCTGTTTTGACCTC	ACGCAGGAGTTTTGATGGAC
MANF $^{fl/fl}$	TGGAGTGAGCACAACTCAGG	CTCAGGTCCTCCACAAGAGC
Umod $^{CRE/+}$	AAGCAGAAAACCTGGTGTGG	ATGCCAACTTGTTTTTGATGG (WT); ACCCCTAGGAATGCTCGTCAAG (Mutant)

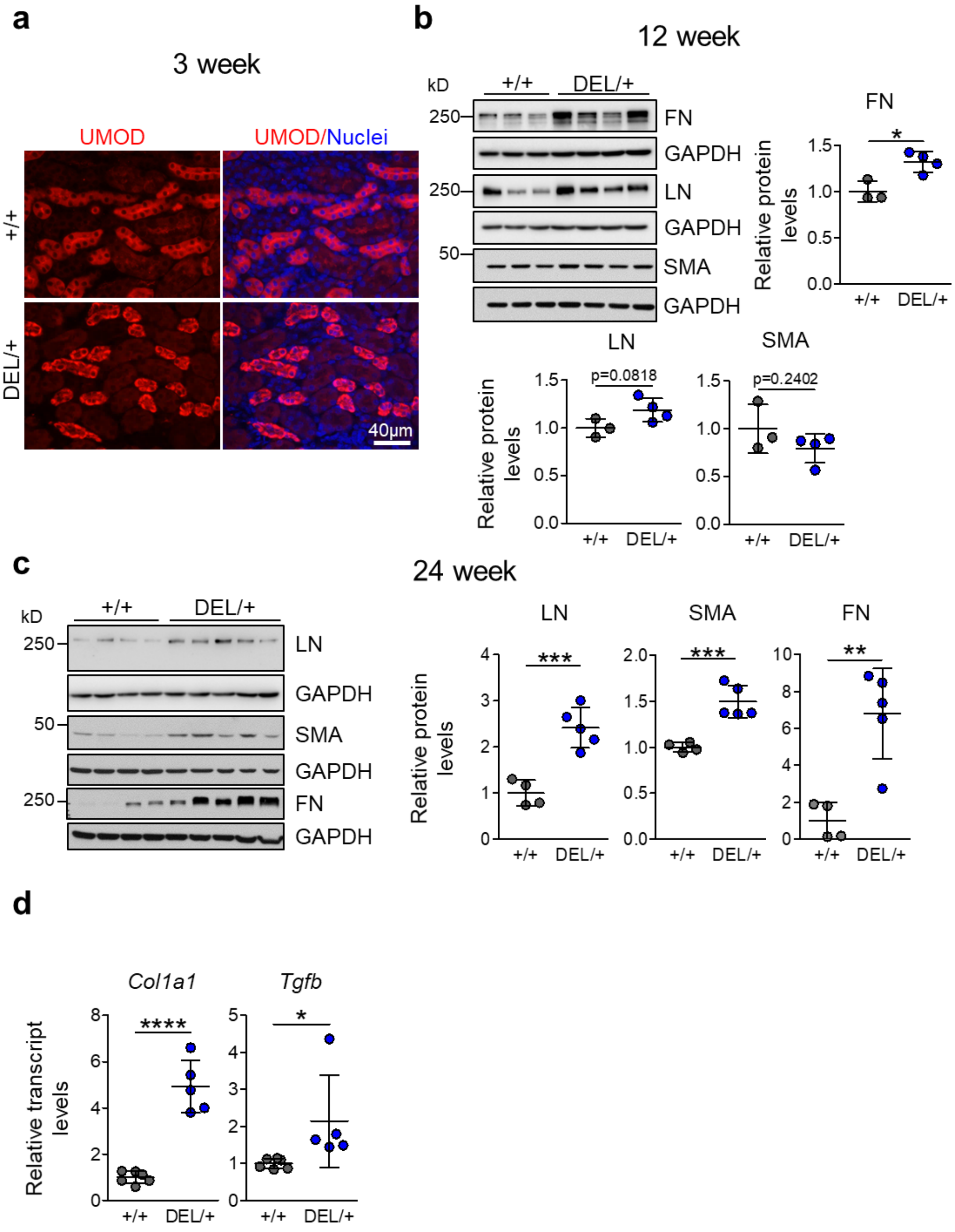

Supplementary Fig. 1 Generation of a mouse model that recapitulates human ADTKD-*UMOD*.

a Representative IF images of paraffin kidney sections stained for UMOD (red) with a nuclear counterstain (Hoechst 33342, blue) at 3 weeks. Scale bar, 40 µm. **b** WBs of whole-kidney lysates from *Umod* $^{+/+}$ ($n = 3$) and *Umod* $^{DEL/+}$ ($n = 4$) mice at 12 weeks to detect FN, LN and SMA with densitometry analysis. Two-tailed *t*-test, Mean ± SD. **p* = 0.0131. **c** Representative immunoblots of whole-kidney lysates from *Umod* $^{+/+}$ ($n = 4$) and *Umod* $^{DEL/+}$ ($n = 5$) mice at 24 weeks to detect LN, SMA and FN with densitometry analysis. Two-tailed *t*-test, Mean ± SD. ****p* = 0.0008 (for LN), ****p* = 0.0009 (SMA), ***p* = 0.0031 (FN). **d** Quantitative RT-PCR analysis of relative transcript levels of *Col1a1* and *Tgfb* in whole kidneys from *Umod* $^{+/+}$ ($n = 6$) and *Umod* $^{DEL/+}$ ($n = 5$) mice at 24 weeks. Gene expression was normalized to 18s. Two-tailed *t*-test, Mean ± SD. *****p* < 0.0001, **p* = 0.0495. Source data are provided as a source data file.

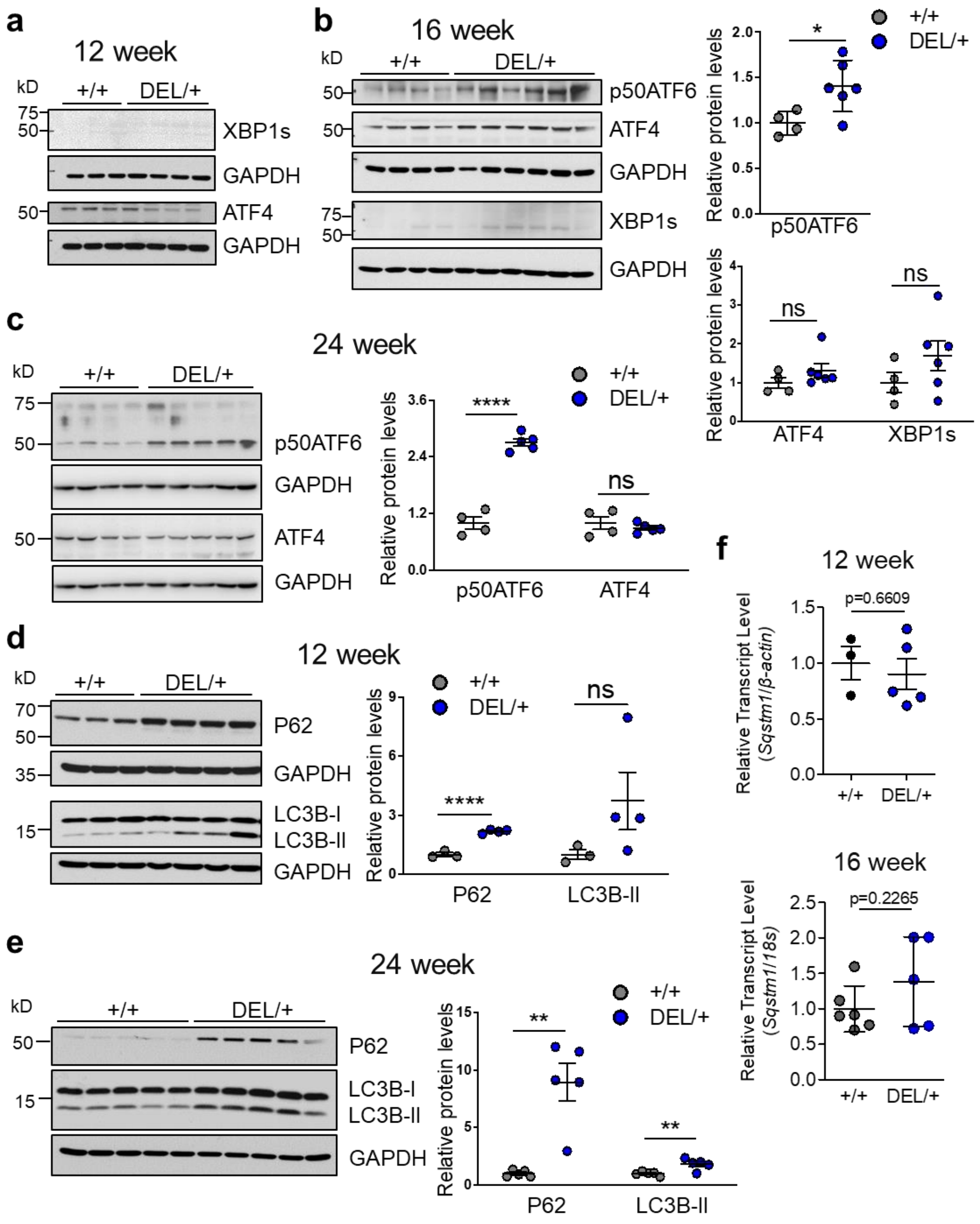

Supplementary Fig. 2 ER stress activation and impaired autophagy in the mouse model of ADTKD-*UMOD*.

a Whole-kidney lysates from *Umod*$^{+/+}$ and *Umod*$^{DEL/+}$ mice at 12 weeks were examined by WBs for XBP1s and ATF4. n = 3-4 mice/genotype. **b** Whole-kidney lysates from *Umod*$^{+/+}$ (n = 4) and *Umod*$^{DEL/+}$ (n = 6) mice at 16 weeks were examined by WBs for p50ATF6, ATF4 and XBP1s with densitometry analysis. Two-tailed t-test, Mean ± SD. *p = 0.0283 (p50ATF6); p = 0.239 (for ATF4, ns, not significant), 0.2136 (for XBP1s, ns). **c** Whole-kidney lysates from *Umod*$^{+/+}$ (n = 4) and *Umod*$^{DEL/+}$ (n = 5) mice at 24 weeks were examined by WBs for p50ATF6 and ATF4 with densitometry analysis. Two-tailed t-test, Mean ± SD. ****p < 0.0001; p = 0.4388 (ns). **d** WB of whole-kidney lysates from *Umod*$^{+/+}$ (n = 3) and *Umod*$^{DEL/+}$ (n = 4) mice to detect the autophagy mediators P62 and LC3B at 12 weeks with densitometry analysis. Two-tailed t-test, Mean ± SD**.** ****p < 0.0001; p = 0.179 (ns). **e** WB of whole-kidney lysates from *Umod*$^{+/+}$ (n = 5) and *Umod*$^{DEL/+}$ (n = 5) mice to detect P62 and LC3B at 24 weeks with densitometry analysis. Two-tailed t-test, Mean ± SD. **p = 0.0012 (for P62), 0.0086 (LC3B-II). **f** Quantitative PCR of *p62* from *Umod*$^{+/+}$ (n = 3, 6 for weeks 12, 16) and *Umod*$^{DEL/+}$ (n = 5, 5 for weeks 12, 16) TALs at 12 and 16 weeks. Two-tailed t-test, Mean ± SD. Source data are provided as a source data file.

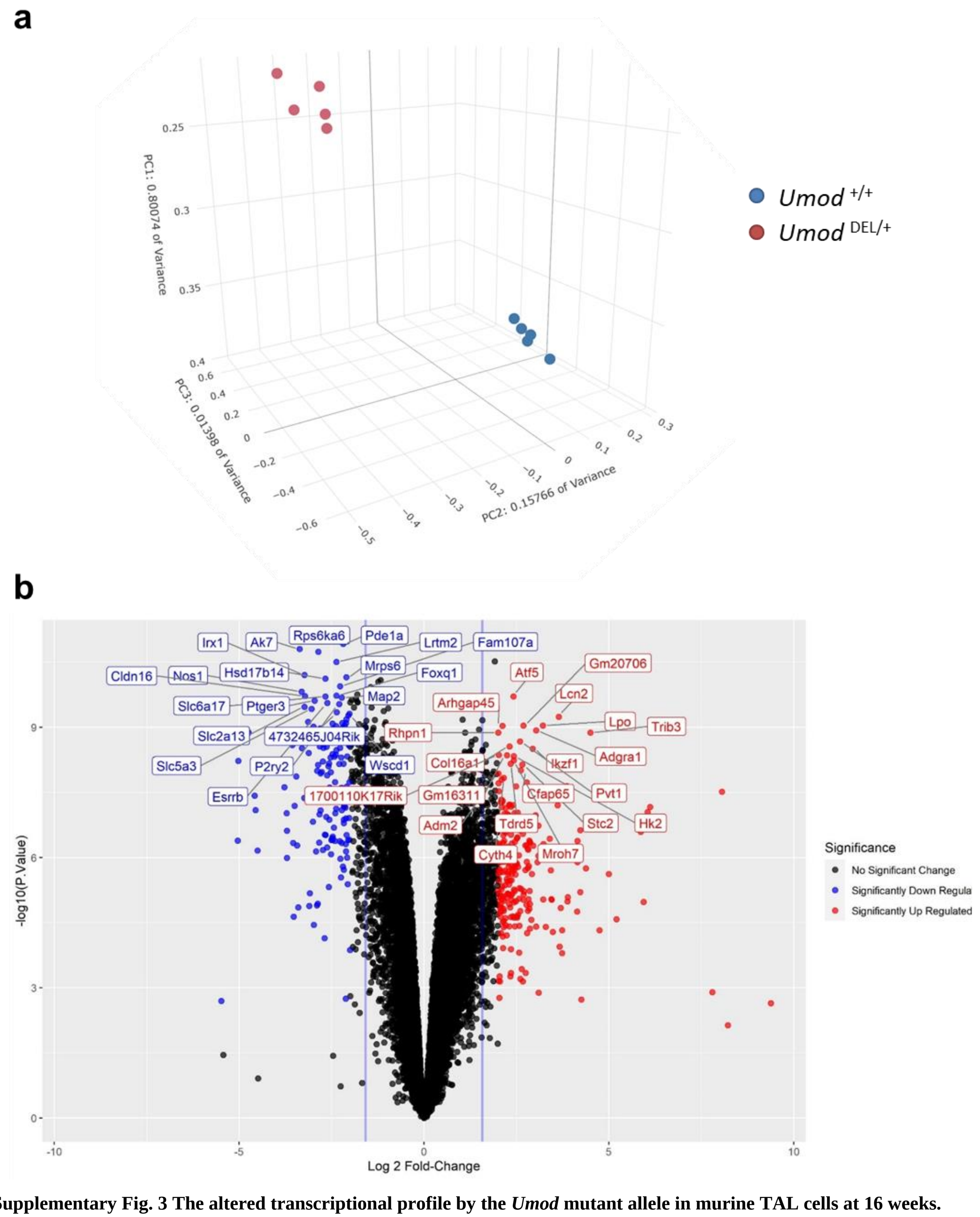

Supplementary Fig. 3 The altered transcriptional profile by the *Umod* mutant allele in murine TAL cells at 16 weeks.

a Multidimensional principal component analysis of RNAseq data obtained from *Umod* $^{DEL/+}$ (n = 5) and WT (n = 5) TALs at 16 weeks. **b** Volcano plot of log2 fold-change and -log10 scale p values, derived by Limma analysis for differential gene expression (Benjamini-Hochberg adjusted p values ≤ 0.05). 9566 were differentially expressed between mutant and WT TAL cells (FDR ≤ 0.05). Out of these transcripts, 244 were up-regulated (red) and 149 were down-regulated (blue) by 4-fold or more. The top 20 most significantly differentiated genes were labeled. Source data are provided as a source data file.

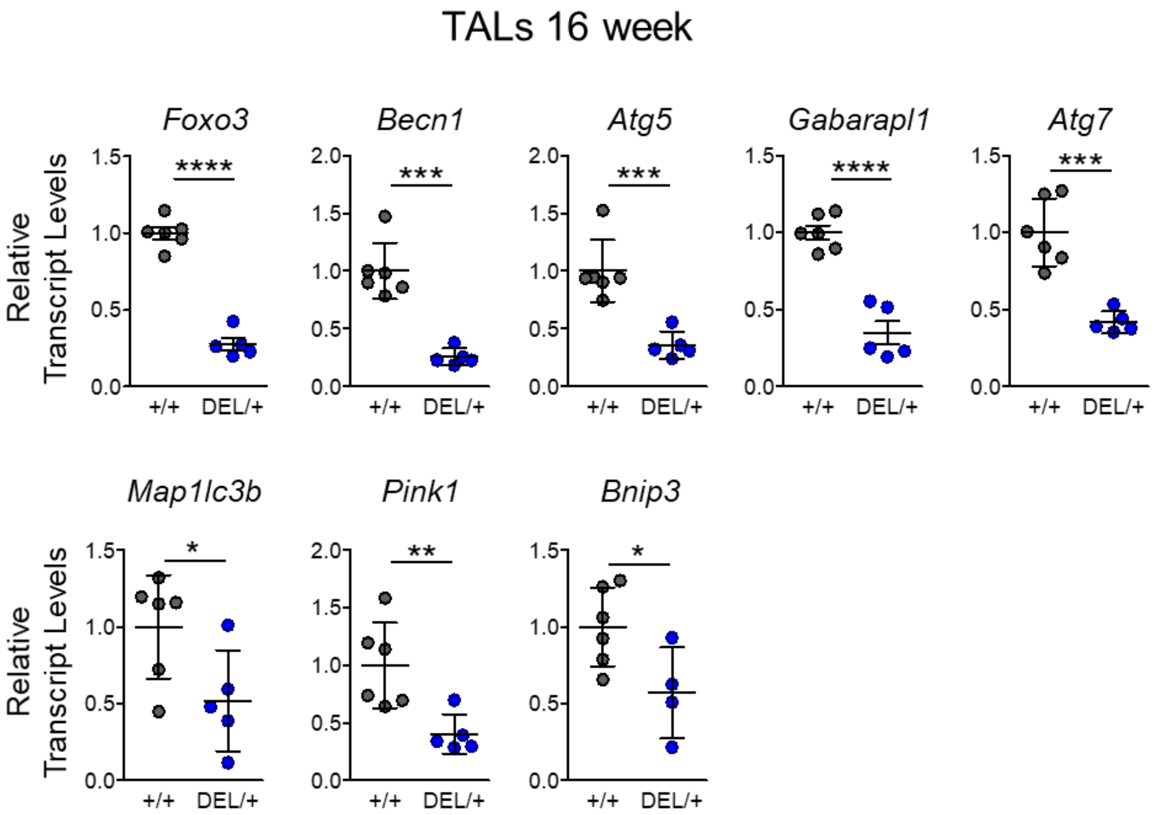

Supplementary Fig. 4 Quantitative PCR validation of RNA-seq results in TAL cells related to autophagy suppression at 16 weeks.

Quantitative PCR of a panel of autophagy-related genes from isolated *Umod* $^{+/+}$ (n = 6) and *Umod* $^{DEL/+}$ (n = 5 except n = 4 for *Bnip3*) TALs at 16 weeks. Gene expression was normalized to 18s. Two-tailed t-test, Mean ± SD. *p = 0.0408 (for *Map1lc3b*), 0.0406 (*Bnip3*); **p = 0.0093 (*Pink1*); ***p = 0.00011 (for *Becn1*), 0.0008 (*Atg5*), 0.0003 (*Atg7*); ****p < 0.0001 (*Foxo3*, *Gabarapl1*). Source data are provided as a source data file.

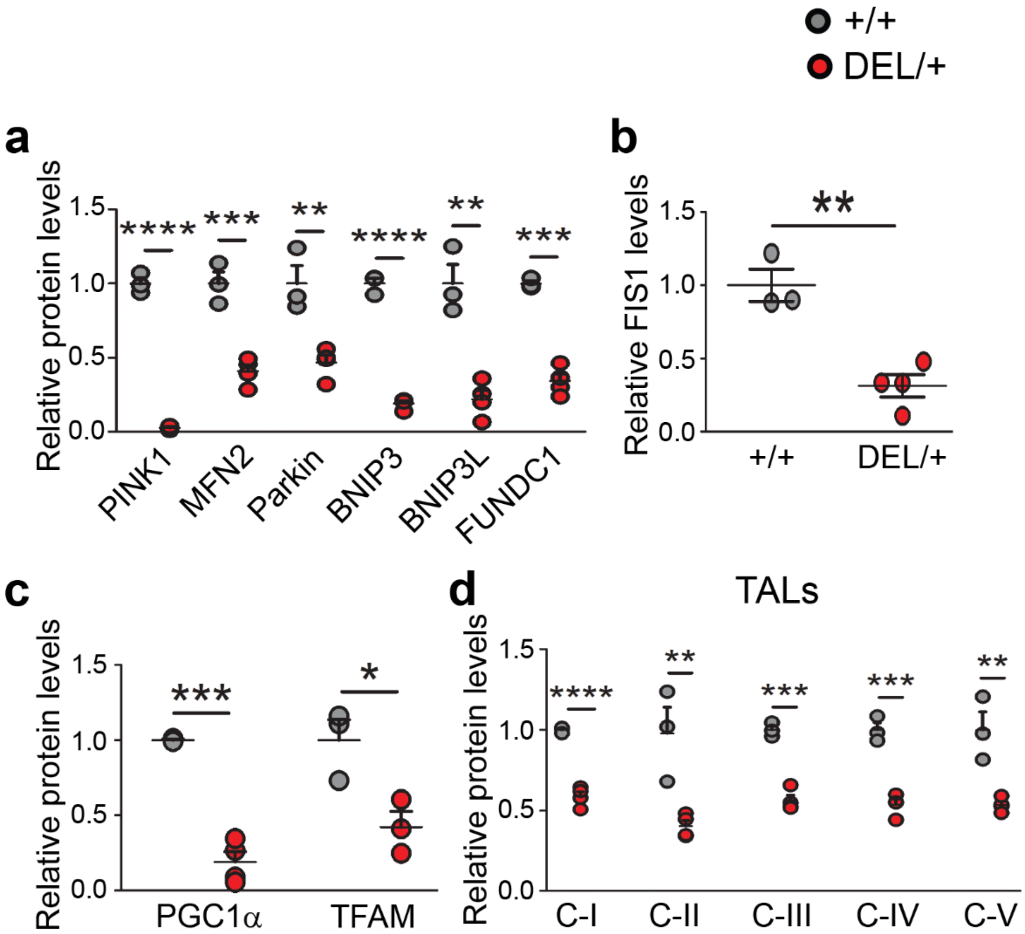

Supplementary Fig. 5 Defective mitophagy, fission and mitochondrial biogenesis in the mutant TALs (densitometry analysis for Fig. 3).

a Densitometry analysis of PINK1, MFN2, Parkin, BNIP3, BNIP3L and FUNDC1 for Fig. 3b. *Umod* $^{+/+}$: $n = 3$, *Umod* $^{DEL/+}$: $n = 4$. Two-tailed *t*-test, Mean ± SD. **$p = 0.0065$ (for Parkin), 0.0018 (BNIP3L); ***$p = 0.00096$ (for MFN2), 0.00011 (FUNDC1); ****$p < 0.0001$ (PINK1, BNIP3). **b** Densitometry analysis of FIS1 for Fig. 3c. *Umod* $^{+/+}$: $n = 3$, *Umod* $^{DEL/+}$: $n = 4$. Two-tailed *t*-test, Mean ± SD. **$p = 0.0031$. **c** Densitometry analysis of PGC1α and TFAM for Fig. 3d. *Umod* $^{+/+}$: $n = 3$, *Umod* $^{DEL/+}$: $n = 4$ (for PGC1α) and $n = 3$ (TFAM). Two-tailed *t*-test, Mean ± SD. *$p = 0.027$; ***$p = 0.0002$. **d** Densitometry analysis of protein expression levels of mitochondrial respiratory complexes I-V for TALs in Fig. 3f. *Umod* $^{+/+}$: $n = 3$, *Umod* $^{DEL/+}$: $n = 4$. Two-tailed *t*-test, Mean ± SD. **$p = 0.0099$ (for C-II), 0.0051 (C-V); ***$p = 0.0002$ (for C-III), 0.0004 (C-IV); ****$p < 0.0001$ (C-I). Source data are provided as a source data file.

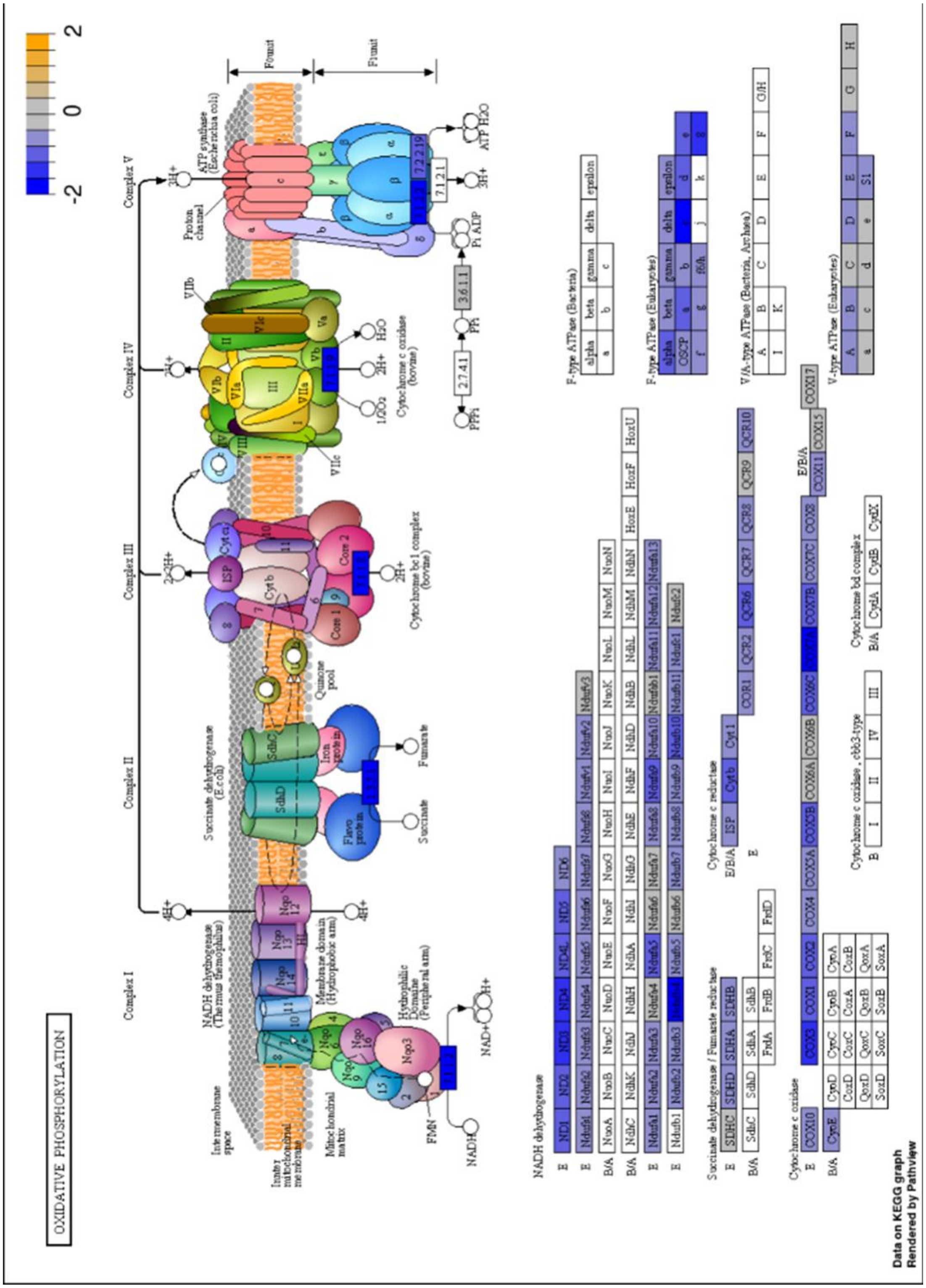

Supplementary Fig. 6 KEGG pathway analysis of mitochondrial respiratory complexes in *Umod* DEL/+ TALs compared with *Umod* +/+ TALs at 16 weeks.

The KEGG pathway depicted downregulation of all five ETCs in the mutant TALs at 16 weeks. Color indicates the magnitude of gene expression changes. The most upregulated genes are highlighted in orange, whereas the most downregulated genes are in blue.

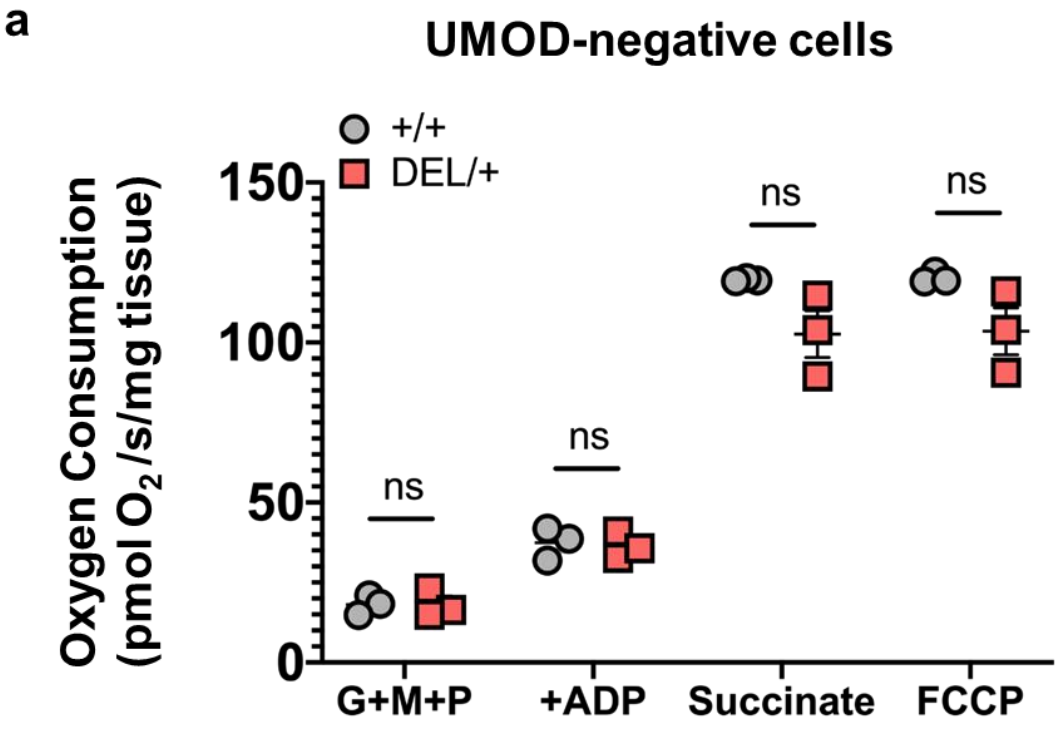

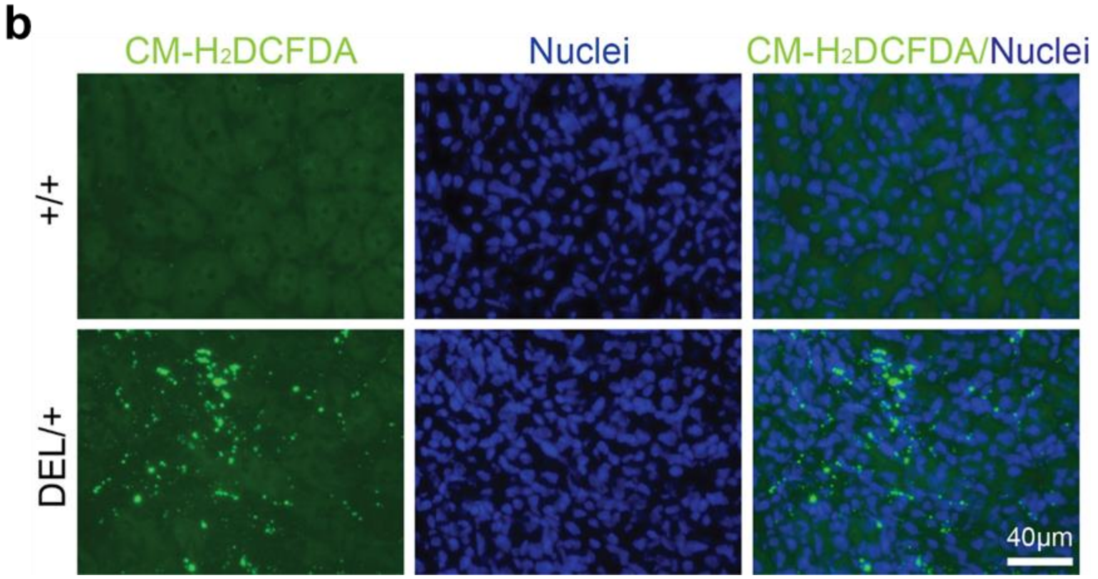

Supplementary Fig. 7 Mitochondrial respiratory function in UMOD^{-} cells and ROS accumulation in *Umod* $^{+/+}$ and *Umod* $^{DEL/+}$ kidneys at 16 weeks.

a Measurement of mitochondrial respiration using an OROBOROS Oxygraph system in permeabilized UMOD-negative cells from *Umod* $^{+/+}$ (n = 3) and *Umod* $^{DEL/+}$ (n = 3) kidneys at 16 weeks following sequential additions of glutamate, malate and pyruvate (G+M+P); adenosine diphosphate (ADP); succinate and FCCP. Two-tailed t-test, Mean ± SD. p = 0.9693 (for G+M+P, ns), 0.802 (ADP, ns), 0.0815 (Succinate, ns), 0.0875 (FCCP, ns). **b** Representative images of ROS staining with nuclei counterstain (blue) on WT and DEL/+ frozen kidney sections at age 16 weeks by utilizing the ROS probe CM-H_2DCFDA. Source data are provided as a source data file.

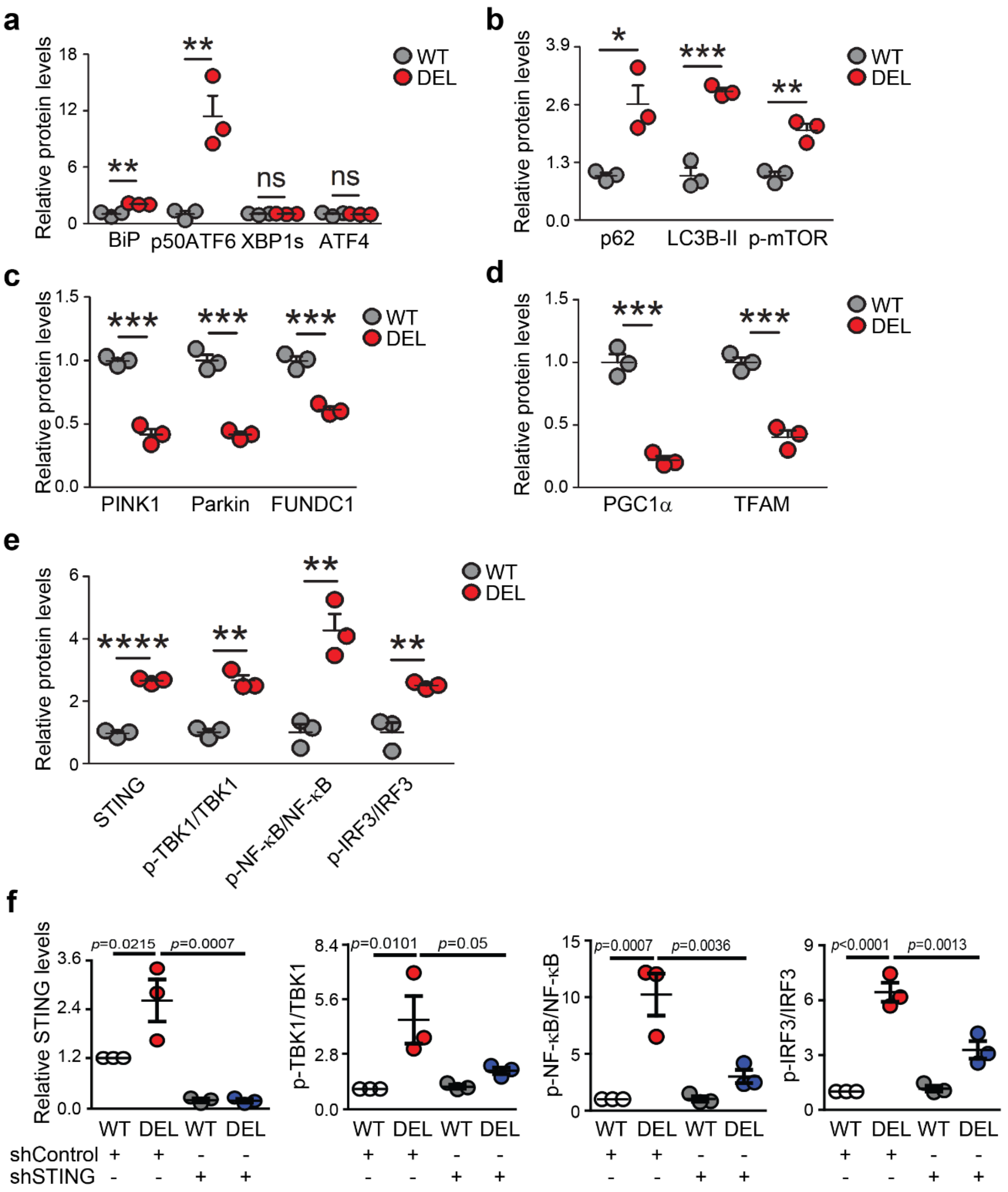

Supplementary Fig. 8 Cytosolic leak of mtDNA activates STING signaling by utilizing stable HEK 293 cell line expressing N-terminal GFP-tagged WT or H177-R185 del UMOD (densitometry analysis for Fig. 5).

a Densitometry analysis of BiP, p50ATF6, XBP1s and ATF4 for Fig. 5e. n = 3/genotype. Two-tailed t-test, Mean ± SD. $^{**}p$ = 0.0028 (for BiP), 0.0093 (p50ATF6); p = 0.8687 (for XBP1s, ns), p = 0.8218 (ATF4, ns). **b** Densitometry analysis of p62, LC3B-II and p-mTOR for Fig. 5f. n = 3/genotype. Two-tailed t-test, Mean ± SD. $^{*}p$ = 0.0187; $^{**}p$ = 0.0037; $^{***}p$ = 0.0006. **c** Densitometry analysis of PINK1, Parkin and FUNDC1 for Fig. 5h. n = 3/genotype. Two-tailed t-test, Mean ± SD. $^{***}p$ = 0.0003 (for PINK1), 0.0003 (Parkin), 0.0009 (FUNDC1). **d** Densitometry analysis of PGC1α and TFAM for Fig. 5i. n = 3/genotype. Two-tailed t-test, Mean ± SD. $^{***}p$ = 0.0004 (for PGC1α), 0.0009 (TFAM). **e** Densitometry analysis of STING, p-TBK1/TBK1, p-NF-κB/NF-κB and p-IRF3/IRF3 for Fig. 5k. n = 3/genotype. Two-tailed t-test, Mean ± SD. $^{**}p$ = 0.0011 (for p-TBK1/TBK1), 0.0049 (p-NF-κB/NF-κB), 0.008 (p-IRF3/IRF3); $^{****}p < 0.0001$ (STING). **f** Densitometry analysis of STING, p-TBK1/TBK1, p-NF-κB/NF-κB and p-IRF3/IRF3 in WT and DEL cells treated with shControl or shSTING for Fig. 5n. n = 3/group. One-way ANOVA with Tukey's multiple comparisons, Mean ± SD. Source data are provided as a source data file.

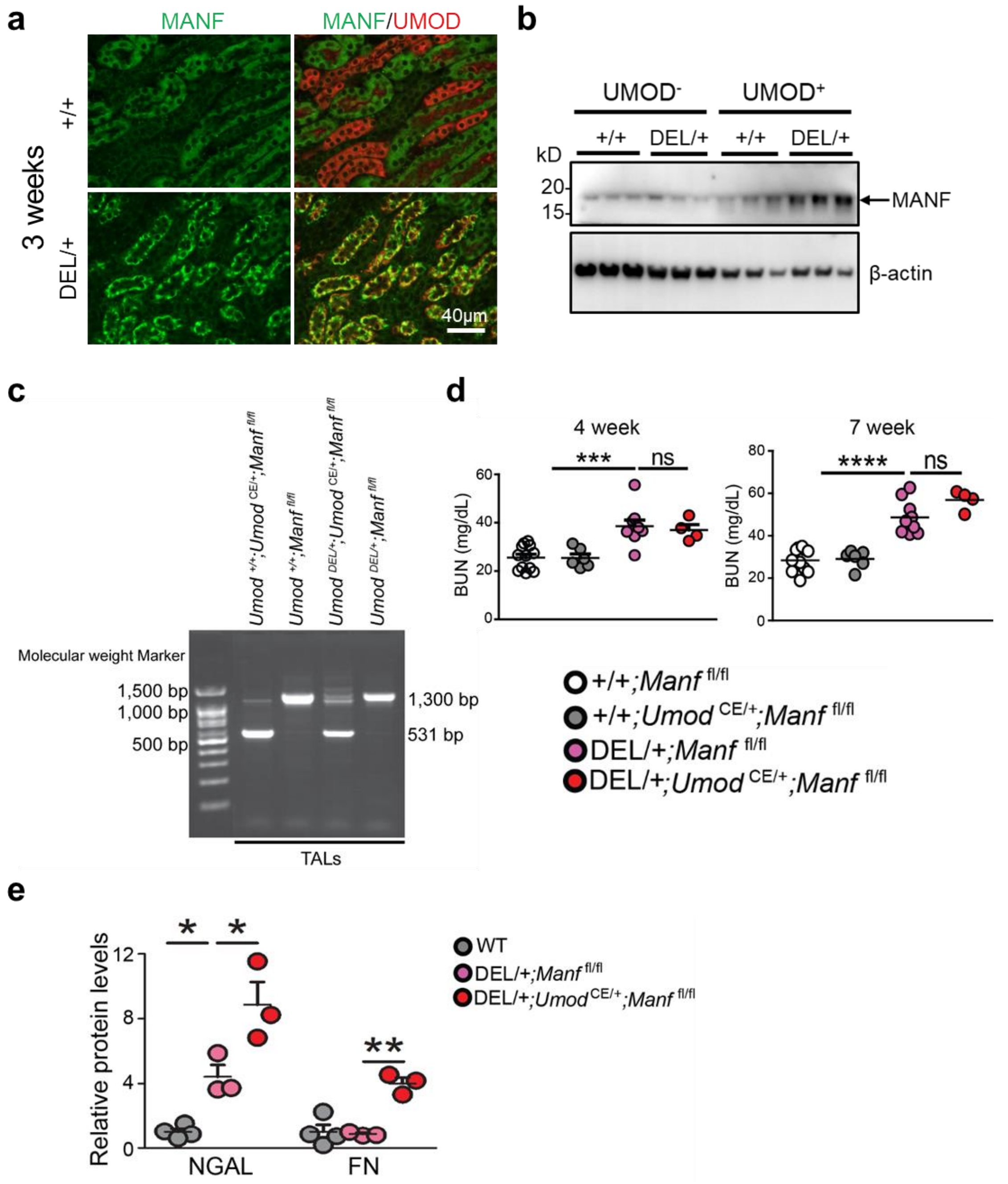

Supplementary Fig. 9 Loss of MANF in TALs deteriorates autophagy suppression and kidney fibrosis in ADTKD.

a Double IF staining for MANF (green) and UMOD (red) on paraffin kidney sections from *Umod* $^{+/+}$ and *Umod* $^{DEL/+}$ mice at 3 weeks of age. Scale bar, 40 µm. **b** WB analysis of MANF in UMOD$^-$ and UMOD$^+$ cells isolated from *Umod* $^{+/+}$ and *Umod* $^{DEL/+}$ mice at 16 weeks of age. *n* = 3 mice/group. **c** PCR results of genomic DNAs from isolated TALs of the indicated genotypes at 12 weeks. Forward primer: 5´-TGG AGT GAG CAC AAC TCA GG-3'; Reverse primer: 5´-TGC CATGGT GAT GCT GTA AC-3´. **d** BUN measurements at 4 and 7 weeks. *Umod* $^{+/+}$;*Manf* $^{fl/fl}$: *n* = 12, *Umod* $^{+/+}$;*Umod* $^{CE/+}$;*Manf* $^{fl/fl}$: *n* = 6, *Umod* $^{DEL/+}$;*Manf* $^{fl/fl}$: *n* = 9, *Umod* $^{DEL/+}$;*Umod* $^{CE/+}$;*Manf* $^{fl/fl}$: *n* = 4. One-way ANOVA with Tukey's multiple comparisons, Mean ± SD. At 4 weeks, ***$p = 0.001$; $p = 0.9687$ (ns); At 7 weeks, ****$p < 0.0001$; $p = 0.1348$ (ns). **e** Densitometry analysis of NGAL and FN for Fig. 6i. WT: *n* = 4, *Umod* $^{DEL/+}$;*Manf* $^{fl/fl}$: *n* = 3, *Umod* $^{DEL/+}$;*Umod* $^{CE/+}$;*Manf* $^{fl/fl}$: *n* = 3. One-way ANOVA with Tukey's multiple comparisons, Mean ± SD. *Umod* $^{DEL/+}$;*Umod* $^{CE/+}$;*Manf* $^{fl/fl}$ vs *Umod* $^{DEL/+}$;*Manf* $^{fl/fl}$: *$p = 0.019$ (NGAL), **$p = 0.0017$ (FN). *Umod* $^{DEL/+}$;*Manf* $^{fl/fl}$ vs WT: *$p = 0.0459$ (NGAL). (**c**-**e**) All mice were given TAM at 5 weeks of age. Source data are provided as a source data file.

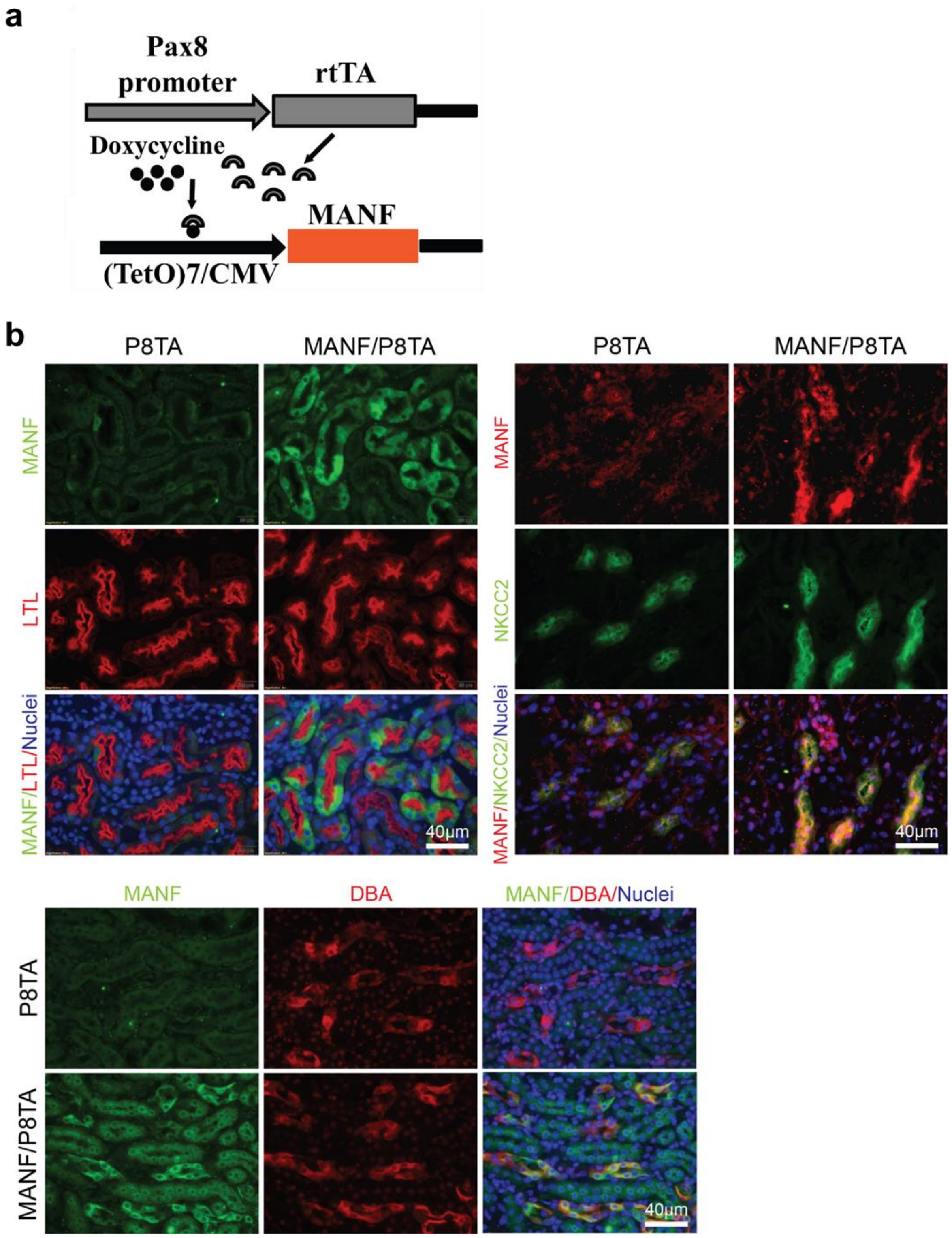

Supplementary Fig. 10 Generation and characterization of inducible, renal tubular MANF transgenic mice.

a Diagram of the two constructs used in the Tg mouse lines for the generation of the Tet-On system. The Pax8 promoter directs expression of rtTA in all renal tubular epithelial cells, and the (TetO)7/CMV-MANF Tg is induced by DOX-bound rtTA. **b** Co-IF staining of MANF and LTL, or NKCC2 or DBA with nuclear counterstain on kidney sections of the indicated groups after 4 weeks of DOX administration. Scale bar, 40 μm.

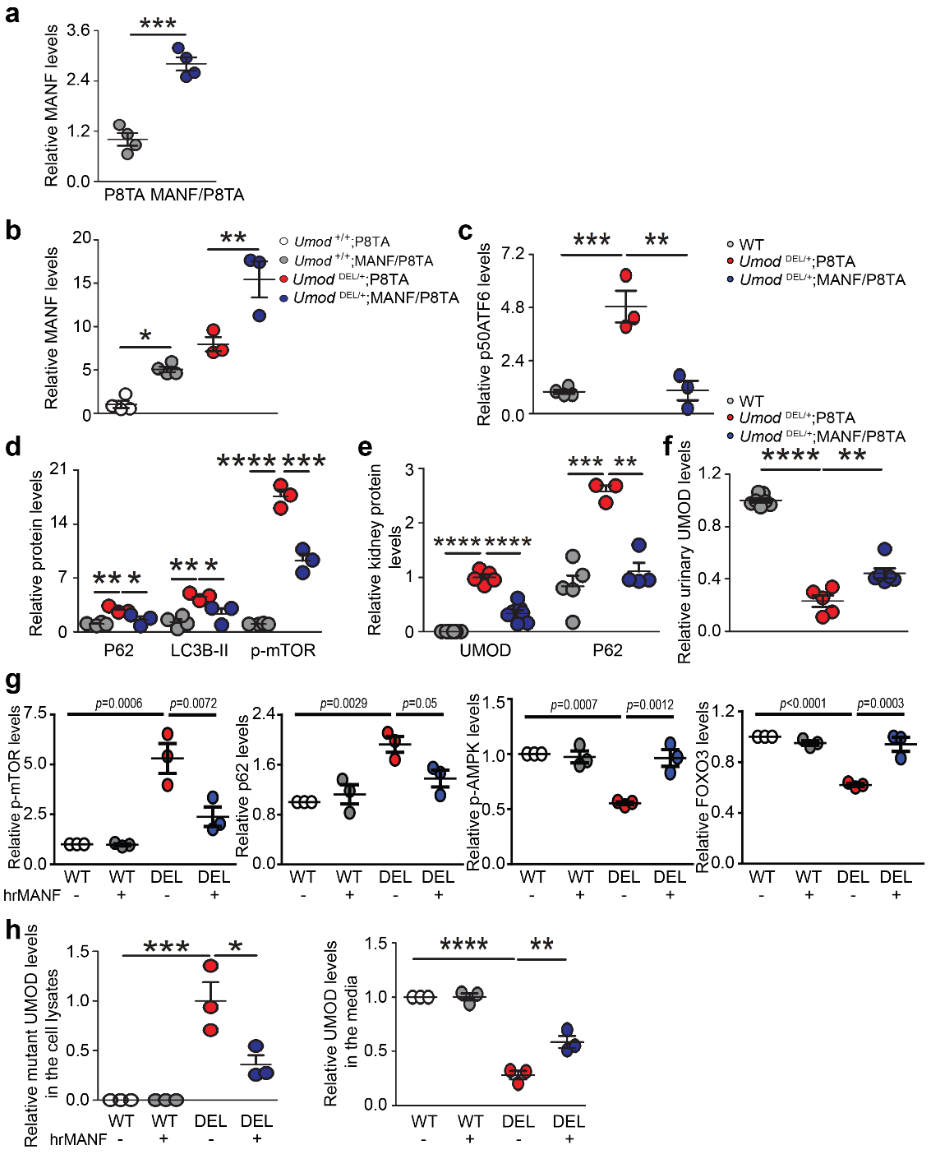

Supplementary Fig. 11 MANF treatment *in vivo* and *in vitro* stimulates autophagy in ADTKD (densitometry analysis for Fig. 7).

a Densitometry analysis of MANF for Fig. 7d. n = 4/genotype. Two-tailed t-test, Mean ± SD. ***p = 0.0002. **b** Densitometry analysis of MANF for Fig. 7f. *Umod* $^{+/+}$;P8TA: n = 4, *Umod* $^{+/+}$;MANF/P8TA: n = 4, *Umod* $^{DEL/+}$;P8TA: n = 3, *Umod* $^{DEL/+}$;MANF/P8TA: n = 3. One-way ANOVA with Tukey's multiple comparisons, Mean ± SD. *p = 0.0433, **p = 0.0025. **c** Densitometry analysis of p50ATF6 for Fig. 7h. WT: n = 4, *Umod* $^{DEL/+}$;P8TA: n = 3, *Umod* $^{DEL/+}$;MANF/P8TA: n = 3. One-way ANOVA with Tukey's multiple comparisons, Mean ± SD. **p = 0.0015; ***p = 0.0009. **d** Densitometry analysis of p62, LC3B-II and p-mTOR for Fig. 7i. WT: n = 4, *Umod* $^{DEL/+}$;P8TA: n = 3, *Umod* $^{DEL/+}$;MANF/P8TA: n = 3. One-way ANOVA with Tukey's multiple comparisons, Mean ± SD. P62: *p = 0.0302, **p = 0.0033; LC3B-II: *p = 0.0356, **p = 0.0039; p-mTOR: ***p = 0.00011; ****p < 0.0001. **e** Densitometry analysis of UMOD and p62 for Fig. 7l. WT: n = 7 (UMOD), n = 5 (P62); *Umod* $^{DEL/+}$;P8TA: n = 5 (UMOD), n = 3 (P62); *Umod* $^{DEL/+}$;MANF/P8TA: n = 7 (UMOD), n = 4 (P62). One-way ANOVA with Tukey's multiple comparisons, Mean ± SD. **p = 0.0012; ***p = 0.0002; ****p < 0.0001. **f** Densitometry analysis of urinary UMOD levels for Fig. 7m. WT: n = 7, *Umod* $^{DEL/+}$;P8TA: n = 5, *Umod* $^{DEL/+}$;MANF/P8TA: n = 6. One-way ANOVA with Tukey's multiple comparisons, Mean ± SD. **p = 0.0013; ****p < 0.0001. **g** Densitometry analysis of p-mTOR, p62, p-AMPK and FOXO3 without or with hrMANF treatment for Fig. 7n. n = 3/group. One-way ANOVA with Tukey's multiple comparisons, Mean ± SD. **h** Densitometry analysis of mutant UMOD levels in the cell lysates or WT UMOD levels in the media in the absence or presence of hrMANF for Fig. 7o. n = 3/group. One-way ANOVA with Tukey's multiple comparisons, Mean ± SD. *p = 0.0124; **p = 0.0019; ***p = 0.0007; ****p < 0.0001. (**a-f**) All mice were given DOX at 4 weeks of age. Source data are provided as a source data file.

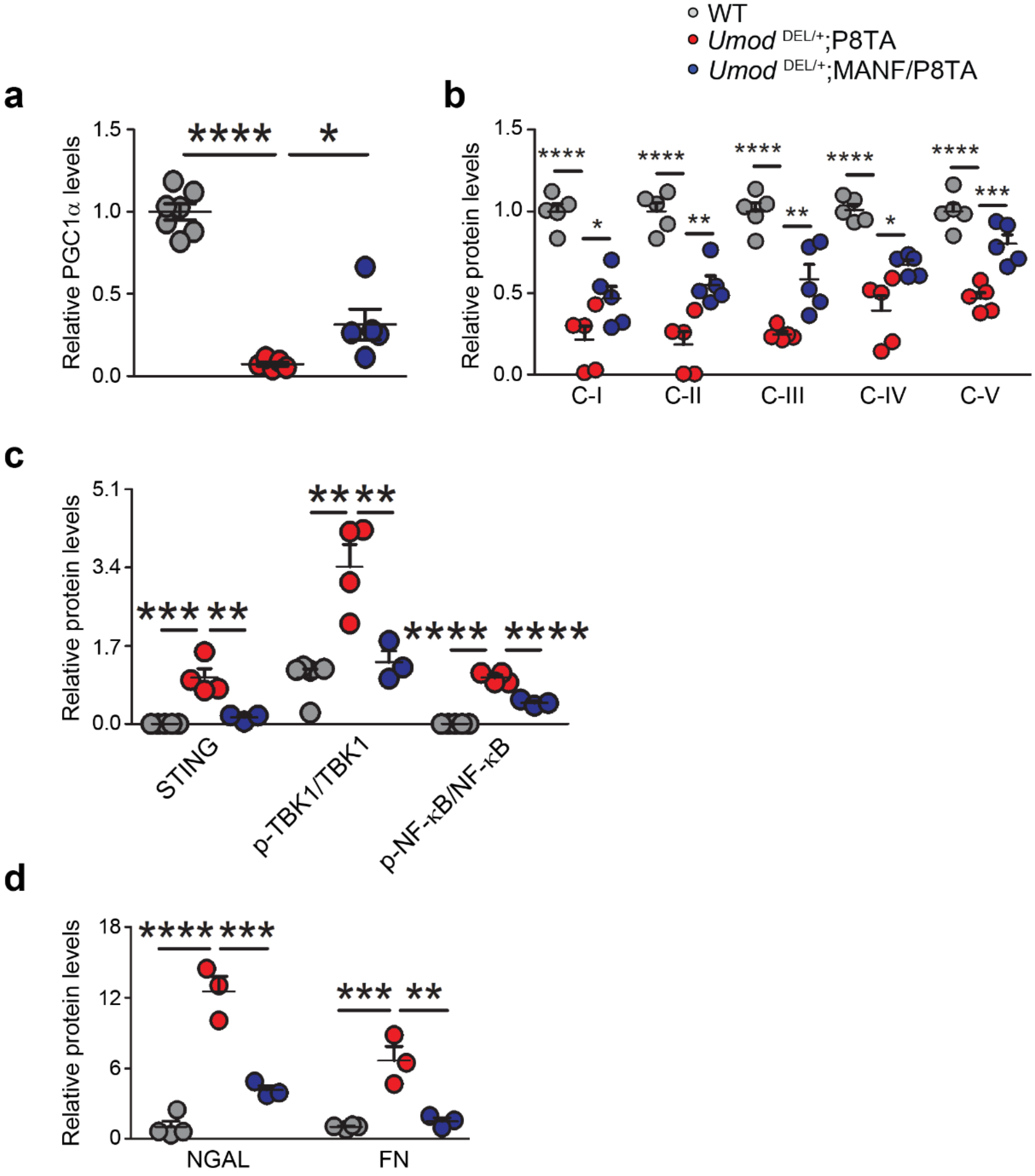

Supplementary Fig. 12 Renal tubular upregulation of MANF promotes mitophagy and improves mitochondrial biogenesis, leading to abrogation of STING activation and kidney fibrosis in ADTKD-*UMOD* (densitometry analysis for Fig. 8).

a Densitometry analysis of PGC1α for Fig. 8b. WT: $n = 7$, *Umod* $^{DEL/+}$;P8TA: $n = 5$, *Umod* $^{DEL/+}$;MANF/P8TA: $n = 5$. One-way ANOVA with Tukey's multiple comparisons, Mean ± SD. *$p = 0.0421$; ****$p < 0.0001$. **b** Densitometry analysis of protein expression levels of mitochondrial respiratory complexes I-V for Fig. 8d. $n = 5$/group. One-way ANOVA with Tukey's multiple comparisons, Mean ± SD. *Umod* $^{DEL/+}$;MANF/P8TA vs *Umod* $^{DEL/+}$;P8TA: $p = 0.0363$ (for C-I), 0.0039 (C-II), 0.0055 (C-III), 0.0132 (C-IV), 0.0009 (C-V). *Umod* $^{DEL/+}$;P8TA vs WT: ****$p < 0.0001$. **c** Densitometry analysis of STING, p-TBK1/TBK1 and p-NF-κB/NF-κB for Fig. 8f. WT: $n = 5$, *Umod* $^{DEL/+}$;P8TA: $n = 4$, *Umod* $^{DEL/+}$;MANF/P8TA: $n = 3$. One-way ANOVA with Tukey's multiple comparisons, Mean ± SD. *Umod* $^{DEL/+}$;MANF/P8TA vs *Umod* $^{DEL/+}$;P8TA: **$p = 0.0021$ (for STING), 0.0066 (p-TBK1/TBK1), ****$p < 0.0001$ (p-NF-κB/NF-κB). *Umod* $^{DEL/+}$;P8TA vs WT: **$p = 0.0011$ (p-TBK1/TBK1), ***$p = 0.0003$ (STING), ****$p < 0.0001$ (p-NF-κB/NF-κB). **d** Densitometry analysis of NGAL and FN for Fig. 8h. WT: $n = 4$, *Umod* $^{DEL/+}$;P8TA: $n = 3$, *Umod* $^{DEL/+}$;MANF/P8TA: $n = 3$. One-way ANOVA with Tukey's multiple comparisons, Mean ± SD. *Umod* $^{DEL/+}$;MANF/P8TA vs *Umod* $^{DEL/+}$;P8TA: **$p = 0.0022$ (FN), ***$p = 0.0004$ (for NGAL). *Umod* $^{DEL/+}$;P8TA vs WT: ***$p = 0.0009$ (FN), ****$p < 0.0001$ (NGAL). (**a-d**) All mice were given DOX at 4 weeks of age. Source data are provided as a source data file.

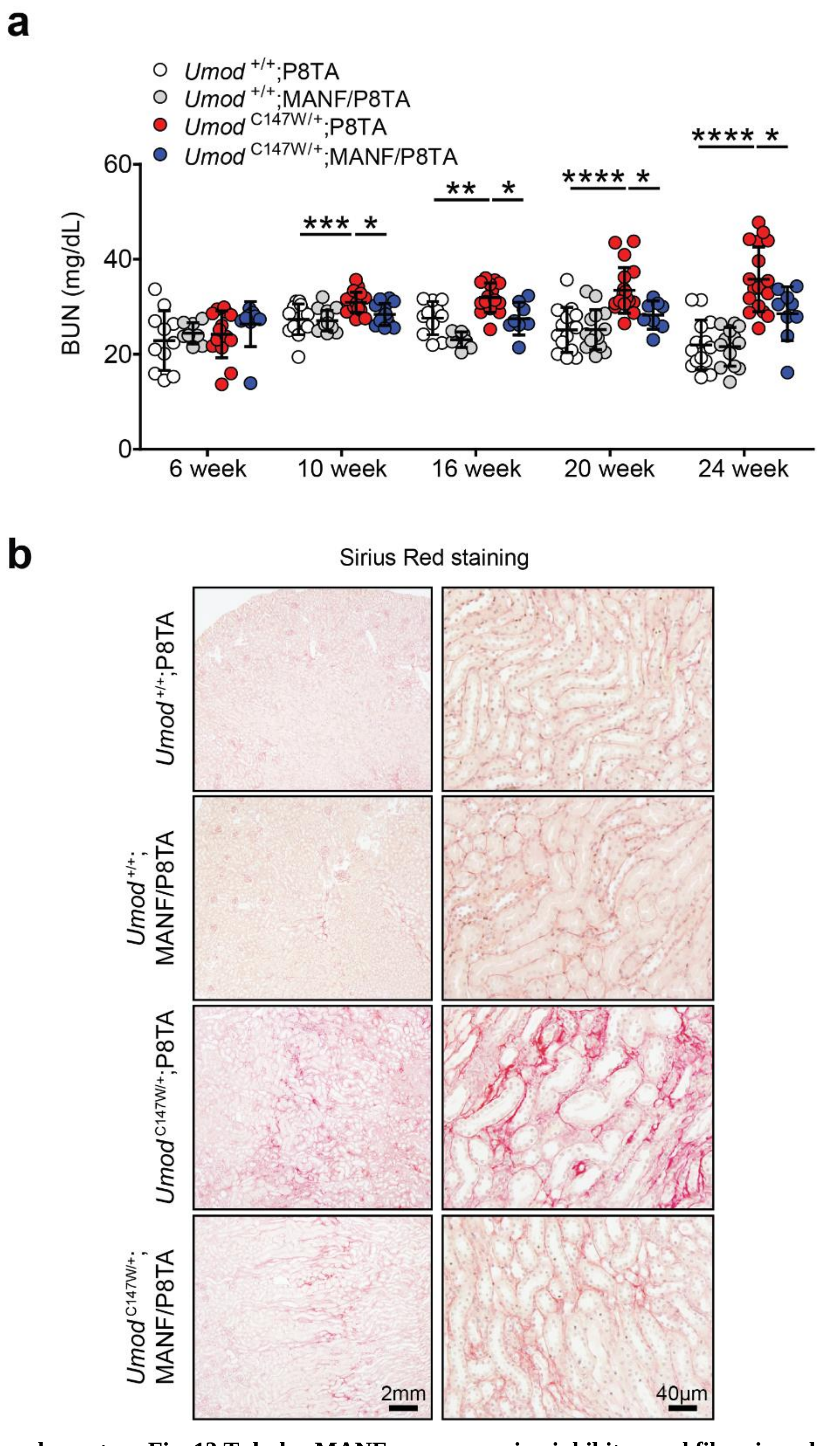

Supplementary Fig. 13 Tubular MANF overexpression inhibits renal fibrosis and protects kidney function in *Umod* $^{C147W/+}$ mice.

a BUN measurements in the indicated groups over a 24-week period. (at 6, 10, 16, 20 and 24 weeks, n = 11, 14, 11, 14, 14 for *Umod* $^{+/+}$;P8TA, n = 8, 16, 8, 14, 13 for *Umod* $^{+/+}$;MANF/P8TA, n = 14, 19, 15, 19, 18 for *Umod* $^{C147W/+}$;P8TA, and n = 9, 13, 8, 8, 9 for *Umod* $^{C147W/+}$;MANF/P8TA mice). One-way ANOVA with Tukey's multiple comparisons, Mean ± SD. *Umod* $^{C147W/+}$;MANF/P8TA vs *Umod* $^{C147W/+}$;P8TA: *p = 0.0298 (for 10 weeks), 0.011 (16 weeks), 0.0382 (20 weeks), 0.0147 (24 weeks). *Umod* $^{C147W/+}$;P8TA vs WT: **p = 0.0055 (16 weeks), ***p = 0.0007 (10 weeks), ****p < 0.0001 (20 and 24 weeks). All mice were given DOX starting from 6 weeks. **b** Representative histological images of whole kidney sections stained with Picrosirius red (collagens I/III in red) at 24 weeks. Scale bar, 2 mm or 40 µm. Source data are provided as a source data file.

Uncropped blots for Supplementary Figure 1b

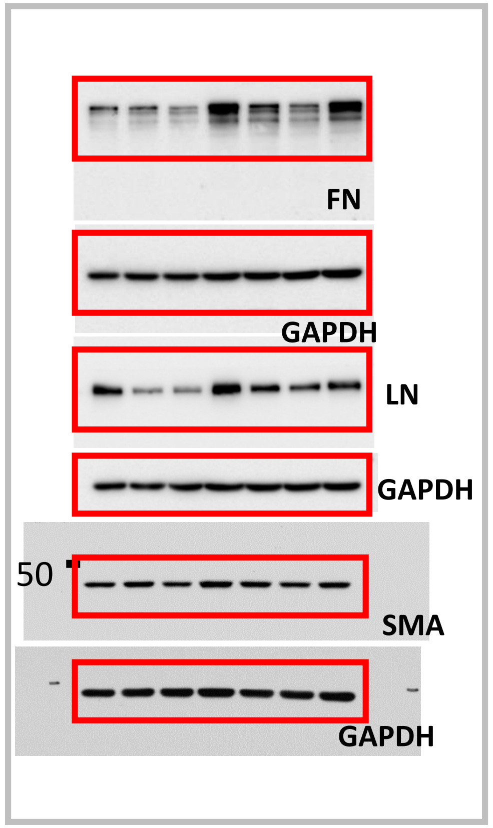

Uncropped blots for Supplementary Figure 1c

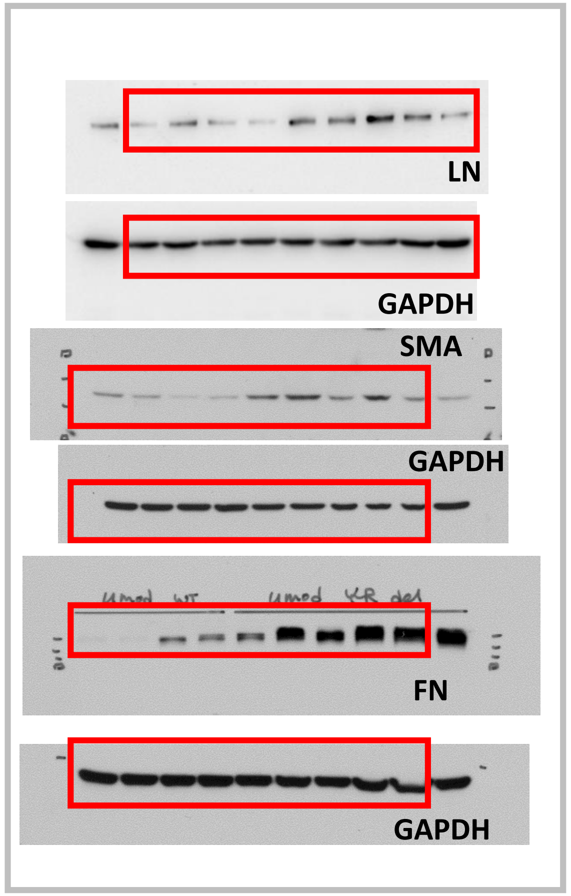

Uncropped blots for Supplementary Figure 2a

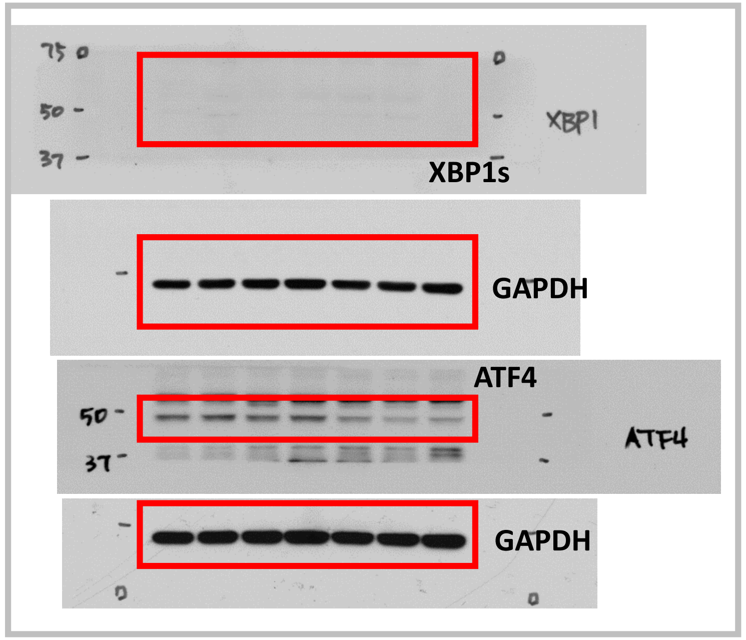

Uncropped blots for Supplementary Figure 2b

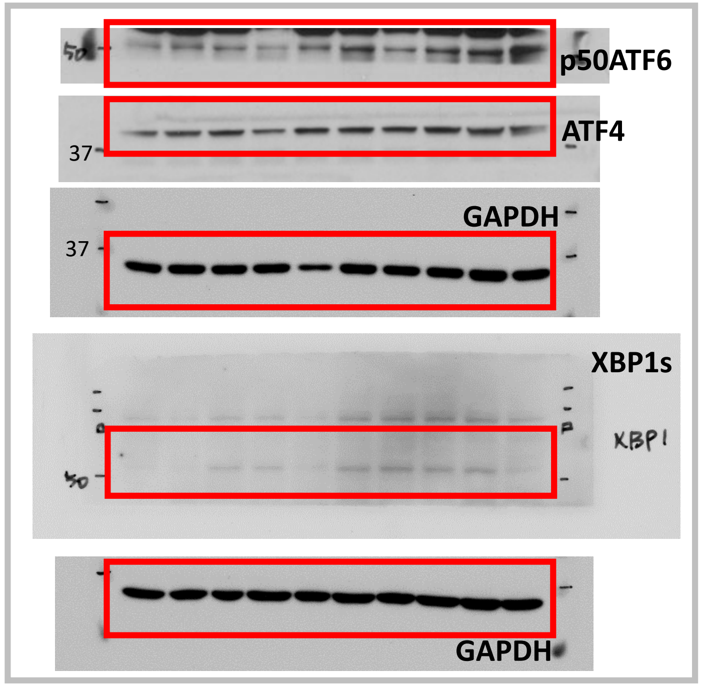

Uncropped blots for Supplementary Figure 2c

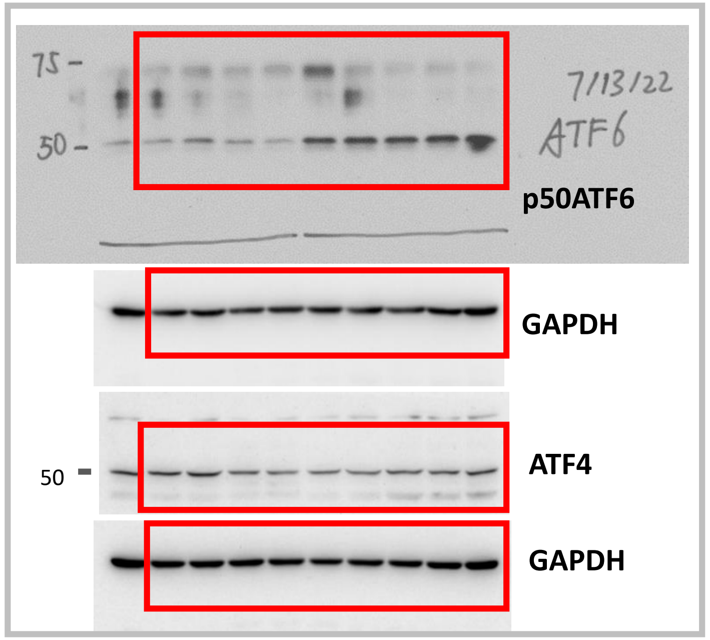

Uncropped blots for Supplementary Figure 2d

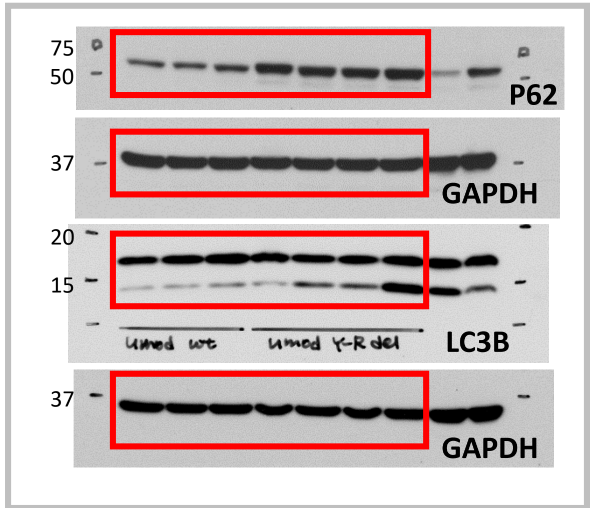

Uncropped blots for Supplementary Figure 2e

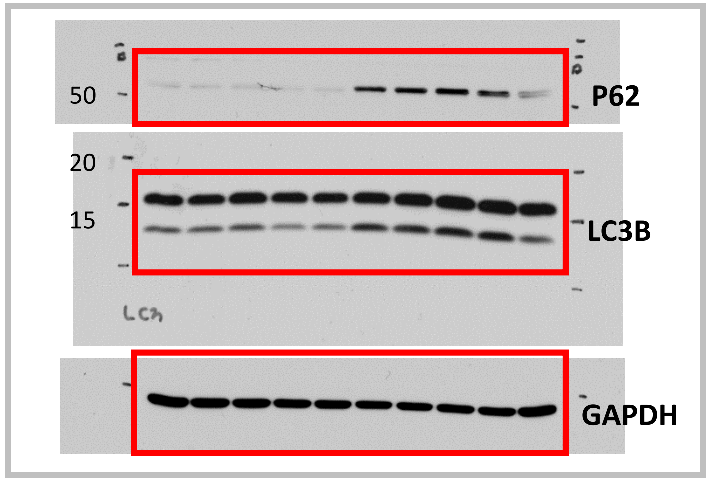

Uncropped blot for Supplementary Figure 9b

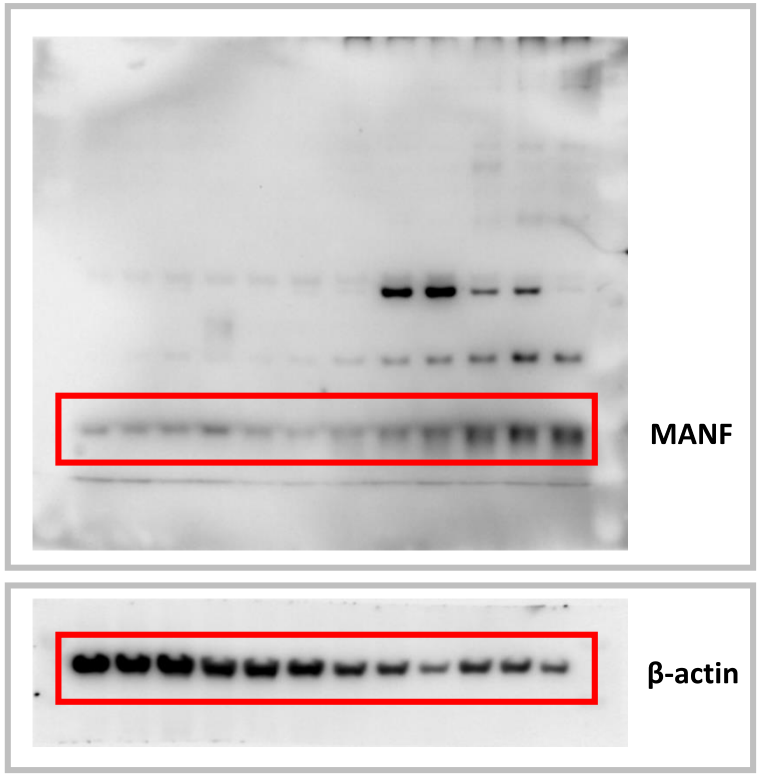

Supplement: Supplementary file 1 — Supplementary Information [file 41467_2023_42154_MOESM1_ESM.pdf]
