# Supplementary material for: MANF stimulates autophagy and restores mitochondrial homeostasis to treat autosomal dominant tubulointerstitial kidney disease in mice
Source: Nat Commun. 2023 Oct 14;14:6493. doi: 10.1038/s41467-023-42154-0 (PMC10576802; doi:10.1038/s41467-023-42154-0)
Supplement: Supplementary file 3 — Reporting Summary [file 41467_2023_42154_MOESM3_ESM.pdf]

nature portfolio

Corresponding author(s): Ying Maggie Chen

Last updated by author(s): Aug. 9, 2023

Reporting Summary

Nature Portfolio wishes to improve the reproducibility of the work that we publish. This form provides structure for consistency and transparency in reporting. For further information on Nature Portfolio policies, see our Editorial Policies and the Editorial Policy Checklist.

Statistics

For all statistical analyses, confirm that the following items are present in the figure legend, table legend, main text, or Methods section.

n/a	Confirmed	
☐	☒	The exact sample size (*n*) for each experimental group/condition, given as a discrete number and unit of measurement
☐	☒	A statement on whether measurements were taken from distinct samples or whether the same sample was measured repeatedly
☐	☒	The statistical test(s) used AND whether they are one- or two-sided<br>*Only common tests should be described solely by name; describe more complex techniques in the Methods section.*
☒	☐	A description of all covariates tested
☐	☒	A description of any assumptions or corrections, such as tests of normality and adjustment for multiple comparisons
☐	☒	A full description of the statistical parameters including central tendency (e.g. means) or other basic estimates (e.g. regression coefficient) AND variation (e.g. standard deviation) or associated estimates of uncertainty (e.g. confidence intervals)
☐	☒	For null hypothesis testing, the test statistic (e.g. *F*, *t*, *r*) with confidence intervals, effect sizes, degrees of freedom and *P* value noted<br>*Give P values as exact values whenever suitable.*
☒	☐	For Bayesian analysis, information on the choice of priors and Markov chain Monte Carlo settings
☒	☐	For hierarchical and complex designs, identification of the appropriate level for tests and full reporting of outcomes
☒	☐	Estimates of effect sizes (e.g. Cohen's *d*, Pearson's *r*), indicating how they were calculated

Our web collection on statistics for biologists contains articles on many of the points above.

Software and code

Policy information about availability of computer code

Data collection	Immunofluorescence staining images were captured by using Nikon 80i Fluorescence Microscope. For live cell imaging, Images were captured using a Nikon Eclipse Ti-E inverted confocal microscope equipped with 10× Plan Fluor (0.30 778 NA), 20× Plan Apo air (0.75 NA), 60× Plan Fluor oil immersion (1.4 NA), or 100× Plan Fluor oil immersion (1.45 NA) objectives (Nikon). cDNA Library preparation was performed with total RNA where the ribosomal RNA was removed by an RNase-H method using RiboErase kits (Kapa Biosystems), then reverse transcribed using SuperScript III RT enzyme. Ligated cDNA fragments were sequenced on an Illumina NovaSeq-6000. Static preclinical PET scans were performed using Inveon PET/CT scanner (Siemens Medical Solutions). For anatomical visualization, PET images were also coregistered with CT images from an Inveon PET/CT scanner. Biodistribution study following Micro PET/CT imaging was checked for gamma-activity using Beckman Gamma 8000 counter. Mitochondrial respirometry was measured using Oxygraph 2K (OROBOROS Instruments, Innsbruck, Austria). Real-time analysis data were collected using QuantStudio 6 Flex Fast Real-Time PCR System (Life Technologies, Grand Island, NY).
Data analysis	Immunofluorescence staining images were analyzed using cellSens standard software (V1.18). Positively stained area was quantified using Image J software (V1.5.3). Live cell images were processed and analyzed using Elements AR 5.21 (Nikon). cDNA fragments reading were performed with Illumina's bcl2fastq software (V2.0). Mitochondrial respirometry was analyzed using the DatLab Software V7.4 (OROBOROS Instruments, Innsbruck, Austria). Real-time data were analyzed using QuantStudio Real-Time PCR software V1.7.1 (Life Technologies). Statistical analyses were performed using GraphPad Prism 8.0 software.<br><br>For RNA sequencing analysis, basecalls and demultiplexing were performed with Illumina's bcl2fastq software (V2.0) and a custom python demultiplexing program with a maximum of one mismatch in the indexing read. RNA-seq reads were then aligned to the Ensembl release 76 primary assembly with STAR version 2.5.1a. Gene counts were derived from the number of uniquely aligned unambiguous reads by Subread:featureCount version 1.4.6-p5. All gene counts were then imported into the R/Bioconductor package EdgeR version 3.32.1 and TMM normalization size factors were calculated to adjust for samples for differences in library size. Ribosomal genes and genes not expressed in at

least 5 samples greater than one count-per-million were excluded from further analysis. The TMM size factors and the matrix of counts were then imported into the R/Bioconductor package Limma version 3.46.0. Weighted likelihoods based on the observed mean-variance relationship of every gene and sample were then calculated for all samples with the voomWithQualityWeights. Differential expression analysis was then performed to analyze for differences between conditions, and the results were filtered for only those genes with Benjamini-Hochberg false-discovery rate (FDR) adjusted p values less than or equal to 0.05.

For each contrast extracted with Limma, global perturbations in known Gene Ontology (GO) terms and KEGG pathways were detected using the R/Bioconductor package GAGE version 2.40.2 to test for changes in expression of the log2 fold-changes reported by Limma in each term versus the background log2 fold-changes of all genes found outside the respective term. GO terms and KEGG pathways with Benjamini-Hochberg adjusted p values less than 0.05 were considered statistically significant. The R/Bioconductor package heatmap3 was used to display heatmaps across groups of samples for each GO with a Benjamini-Hochberg FDR adjusted p value less than or equal to 0.05.

For manuscripts utilizing custom algorithms or software that are central to the research but not yet described in published literature, software must be made available to editors and reviewers. We strongly encourage code deposition in a community repository (e.g. GitHub). See the Nature Portfolio guidelines for submitting code & software for further information.

Data

Policy information about availability of data

All manuscripts must include a data availability statement. This statement should provide the following information, where applicable:

- Accession codes, unique identifiers, or web links for publicly available datasets
- A description of any restrictions on data availability
- For clinical datasets or third party data, please ensure that the statement adheres to our policy

All data needed to evaluate the conclusions in the paper are present in the paper and/or the Supplementary Materials. The generated raw fastq sequencing files and gene counts data in this study needed for reproducibility with publicly available tools cited in the Methods have been deposited in Gene Expression Omnibus [GEO] database, accession no. GSE 230005 (https://www.ncbi.nlm.nih.gov/geo/query/acc.cgi?acc=GSE230005). Source data are provided with this paper.

The analysis code for the RNA-seq data uses all published and publicly available tools and algorithms as cited in the Methods and only requires familiarity with command line interfaces in a Linux equivalent operating system and the R statistical computing language. All code to reproduce the RNA-seq results is maintained by the Washington University Genome Technology Access Center at The McDonnell Genome Institute (GTAC@MGI) and is under restricted access due to the fee for service design of the core facility. Please email the GTAC@MGI bioinformatics at GTAC-Bioinformatics@path.wustl.edu for access to code and docker computing environments containing the RNA-seq tools cited in the Methods, which can be made available within 48 hours upon request after a 24 hour response time during normal business hours.

Human research participants

Policy information about studies involving human research participants and Sex and Gender in Research.

Reporting on sex and gender	N/A
Population characteristics	N/A
Recruitment	N/A
Ethics oversight	All human paraffin-embedded slides from ADTKD patients were obtained from the Wake Forest Cohort under the protocol approved by the Institutional Review Board of Wake Forest School of Medicine. The written informed consent was obtained from the patients. The slides are stored and provided for the current study in a de-identified manner.

Note that full information on the approval of the study protocol must also be provided in the manuscript.

Field-specific reporting

Please select the one below that is the best fit for your research. If you are not sure, read the appropriate sections before making your selection.

☒ Life sciences ☐ Behavioural & social sciences ☐ Ecological, evolutionary & environmental sciences

For a reference copy of the document with all sections, see nature.com/documents/nr-reporting-summary-flat.pdf

Life sciences study design

All studies must disclose on these points even when the disclosure is negative.

Sample size	For cell-based experiments, n = 3-4 per group was chosen, and at least two-three times were repeated for each assay. The sample size was chosen to produce statistically significant biological difference in the study.<br><br>For animal studies, specific sample size for the relevant experiment is shown in the figure legend, which indicates "n" number for each group. The sample size was determined based on the minimal number of animals being sacrificed while at the same time, fulfilling statistically.

Statistical analysis was performed using Graphpad Prism 8.0 software. All statistical information is described in the figure legends. In general, comparisons between two groups are performed with two-tailed t-test, and multiple group comparisons were performed by one-way ANOVA with post-hoc Tukey test.

Data exclusions	No data were excluded.
Replication	At least three independent biological repeats were performed for all experiments. The major findings were cross-validated using multiple and different methodologies (RNA-sequencing, immunofluorescence staining, qPCR, western blot, PET/CT and high resolution respirometry).
Randomization	For in vivo studies, mice were randomly allocated into experimental groups based on their genotypes. The breeding was set up in order to obtain the desired genotypes in the same litter, and littermates were used for each experiment. All cells were allocated in random. No bias in sample allocation was involved.
Blinding	Masson's Trichrome staining was quantified by 2 blinded researchers.<br>For all other experiments not listed above, blinding was not conducted during experiments because data reported for cell and mouse experiments were not subjective but rather based on quantitative analyses, which is not influenced by investigator's bias.

Reporting for specific materials, systems and methods

We require information from authors about some types of materials, experimental systems and methods used in many studies. Here, indicate whether each material, system or method listed is relevant to your study. If you are not sure if a list item applies to your research, read the appropriate section before selecting a response.

Materials & experimental systems

n/a	Involved in the study	
☐	☒	Antibodies
☐	☒	Eukaryotic cell lines
☒	☐	Palaeontology and archaeology
☐	☒	Animals and other organisms
☒	☐	Clinical data
☒	☐	Dual use research of concern

Methods

n/a	Involved in the study	
☒	☐	ChIP-seq
☒	☐	Flow cytometry
☒	☐	MRI-based neuroimaging

Antibodies

Antibodies used

All antibodies and associated information listed in Method are also provided below:

mouse monoclonal anti-human & mouse β-actin-Peroxidase (Sigma, A3854, Clone: AC-15, Lot: 089M4850V), 1:20,000 (WB)
rabbit monoclonal anti-human & mouse AMPKα (Cell Signaling, 5832, Clone: D63G4, Lot:5), 1:1,000 (WB)
rabbit monoclonal anti-human & mouse Phospho AMPKα (Thr172) (Cell Signaling, 50081, Clone: D4D6D, Lot: 3), 1:1,000 (WB)
rabbit polyclonal anti-human & mouse ATF4 (Santa Cruz, sc-200, Lot:I2415), 1:1,000 (WB)
mouse monoclonal anti-human & mouse ATF6 (NOVUS biochemicals, NBP1-40256, Clone: 70B1413.1, Lot:F-3) 1:1,000 (WB)
rabbit polyclonal anti-mouse BIP (Proteintech, 11587-1-AP, Lot # not available) 1:1,000 (WB), 1:50 (IF)
rabbit anti-mouse BNIP3 (Cell Signaling, 3769, Lot:2), 1:1,000 (WB)
rabbit monoclonal anti-mouse BNIP3L/Nix (Cell Signaling, 12396, Clone: D4R4B, Lot:2), 1:1,000 (WB)
rabbit polyclonal anti-Calnexin (Santa Cruz, sc-11397, Lot:G1910), 1:50 (IF)
rabbit anti-human & mouse Caspase 3 (Cell Signaling, 9662, Lot:19), 1:1,000 (WB)
mouse monoclonal anti-human & mouse Caspase 9 (Cell Signaling, 9508, Clone: C9, Lot:6), 1:1,000 (WB)
rabbit monoclonal anti-human & mouse cleaved caspase 3 (Cell Signaling, 9664, Clone: 5A1E, Lot:22), 1:1,000 (WB)
rabbit polyclonal anti-mouse Collagen I (Abcam, ab34710, Lot:GR174212-2), 1:50 (IF)
rabbit anti-mouse COX IV (Cell Signaling, 4844, Lot:3), 1:1,000 (WB)
goat polyclonal anti-mouse CRELD2 (R&D Systems, AF3686, Lot:XXQ016081), 1:1,000 (WB)
rat monoclonal anti-mouse F4/80 (Invitrogen, 14-4801-82, Clone: BM8, Lot:2488480), 1:50 (IF)
rabbit polyclonal anti-mouse Fibronectin (Abcam, ab2413, Lot:GR3411873-1), 1:1,000 (WB), 1:50 (IF)
rabbit polyclonal anti-mouse FIS1 (GeneTex, GTX111010, Lot:42795), 1:1,000 (WB)
rabbit monoclonal anti-human & mouse Foxo3a (Cell Signaling, 12829, Clone: D19A7, Lot:6), 1:1,000 (WB)
rabbit monoclonal anti-human & mouse FUNDC1 (Cell Signaling, 49240, Clone: E2F4T, Lot:1), 1:1,000 (WB)
rabbit monoclonal anti-human & mouse GAPDH (Cell Signaling, 5174, Clone: D16H11, Lot:8), 1:5,000 (WB)
mouse anti-GFP (Roche, 11814460001, Clone:7.1 & 13.1) 1:1,000 (WB)
rabbit monoclonal anti-human & mouse IRF3 (Cell Signaling, 4302, Clone: D83B9, Lot:7) 1:1,000 (WB)
rabbit monoclonal anti-human & mouse phospho-IRF3 (Ser396) (Cell Signaling, 29047, Clone: D6O1M, Lot:4), 1:500 (WB)
rabbit polyclonal anti-mouse Laminin (Abcam, ab11575, Lot:GR3426862-2), 1:2,000 (WB)
rabbit anti-human & mouse LC3A/B (Cell Signaling, 4108, Lot:13), 1:1,000 (WB)
rabbit polyclonal anti-human & mouse MANF (Abnova, PAB13301, Lot:8922-1903), 1:1,000 (WB), 1:100 (IF)
goat polyclonal anti-human MANF (R&D Systems, AF3748, Lot: YLB032303A), 1:50 (IF)
rabbit monoclonal anti-mouse Mitofusin2 (Cell Signaling, 9482, Clone: D2D10, Lot:4), 1:1,000 (WB)
rabbit anti-human & mouse Phospho mTOR (Ser2481) (Cell Signaling, 2974, Lot:12), 1:1,000 (WB)
rabbit polyclonal anti-human & mouse NF-kB p65 (Abcam, ab16502, Lot:GR3382816-2), 1:1,000 (WB)
rabbit monoclonal anti-human & mouse phospho-NF-kB p65 (Ser536) (Cell Signaling, 3033, Clone: 93H1, Lot:19), 1:500 (WB)

goat polyclonal anti-mouse NGAL (R&D Systems, AF1857, Lot:JZP0521011), 1:1,000 (WB)
rabbit anti-mouse NKCC2 (Alpha Diagnostic, NKCC21-A, Lot:427778A3.3-P) 1:50 (IF)
rodent OXPHOS antibody cocktail (Abcam, ab110413, Lot:2101006616), 1:2,000 (WB)
rabbit anti-human & mouse p62/SQSTM1 (Cell Signaling, 5114, Lot:6), 1:1,000 (WB), 1:50 (IF)
mouse monoclonal anti-human & mouse Parkin (Abcam, ab77924, Clone: PRK8, Lot:1013226-1), 1:1,000 (WB)
mouse monoclonal anti-human & mouse PGC1a (Proteintech, 66369-1-AP, Clone: 1C1B2, Lot:10018763), 1:1,000 (WB)
rabbit polyclonal anti-human & mouse PINK1 (Abcam, ab23707, Lot:GR3375868-1), 1:1,000 (WB)
mouse monoclonal anti-mouse Smooth muscle actin (Sigma, A2547, Clone: 1A4, Lot:128M4481V), 1:1,000 (WB), 1:50 (IF)
rabbit monoclonal anti-human STING (Cell Signaling, 13647, Clone: D2P2F, Lot:9), 1:1,000 (WB)
rabbit monoclonal anti-mouse STING (Cell Signaling, 50494, Clone: D1V5L, Lot:1), 1:1,000 (WB)
rabbit monoclonal anti-human & mouse TBK1/NAK (Cell Signaling, 3504, Clone: D1B4, Lot:13), 1:1,000 (WB)
rabbit monoclonal anti-human & mouse phospho-TBK1/NAK (Ser172) (Cell Signaling, 5483, Clone: D52C2, Lot:5), 1:1,000 (WB)
rabbit anti-human & mouse TFAM (Sigma, SAB1401383, Lot:K9211) 1:1,000 (WB)
rat monoclonal anti-mouse Uromodulin (R&D Systems, MAB5175, Clone: 774056, Lot:CGTD0119121), 1:1,000 (WB), 1:50 (IF)
rabbit anti-mouse Uromodulin (Alfa Aesar, J65429, Lot:590D19A) 1:1,000 (WB)
sheep polyclonal anti-mouse Uromodulin, Biotin (R&D Systems, BAF5175, Lot:CBJQ0110051), 4ug per mouse (TAL cell isolation)
mouse monoclonal anti-human Uromodulin (RayBiotech, 119-13298, Clone: 10.32, Lot:203880RAY8) 1:1,000 (WB), 1:50 (IF)
rabbit polyclonal anti-human & mouse XBP1s (BioLegend, 619502, Lot:B205853), 1:1,000 (WB)

Validation

All antibodies listed above are commercially available and validated by correspondent suppliers, which is described in the manufacturer's website.

- mouse anti-human & mouse β-actin-Peroxidase antibody is validated for WB: https://www.sigmaaldrich.com/US/en/product/sigma/a3854
- rabbit anti-human & mouse AMPKα antibody is validated for WB: https://www.cellsignal.com/products/primary-antibodies/ampka-d63g4-rabbit-mab/5832
- rabbit anti-human & mouse Phospho AMPKα (Thr172) antibody is validated for WB: https://www.cellsignal.com/products/primary-antibodies/phospho-ampka-thr172-d4d6d-rabbit-mab/50081
- rabbit anti-human & mouse ATF4 antibody is validated for WB: https://www.scbt.com/p/creb-2-antibody-c-20
- mouse anti-human & mouse ATF6 antibody is validated for WB: https://www.novusbio.com/products/atf6-antibody-70b14131_nbp1-40256
- rabbit anti-mouse BIP antibody is validated for WB & IF: https://www.ptglab.com/products/GRP78,BIP-Antibody-11587-1-AP.htm
- rabbit anti-mouse BNIP3 antibody is validated for WB: https://www.cellsignal.com/products/primary-antibodies/bnip3-antibody-rodent-specific/3769
- rabbit anti-mouse BNIP3L/Nix antibody is validated for WB: https://www.cellsignal.com/products/primary-antibodies/bnip3l-nix-d4r4b-rabbit-mab/12396
- rabbit anti-human Calnexin antibody is validated for IF: https://www.scbt.com/p/calnexin-antibody-h-70
- rabbit anti-human & mouse Caspase 3 antibody is validated for WB: https://www.cellsignal.com/products/primary-antibodies/caspase-3-antibody/9662
- mouse anti-human & mouse Caspase 9 antibody is validated for WB: https://www.cellsignal.com/products/primary-antibodies/caspase-9-c9-mouse-mab/9508
- rabbit anti-human & mouse Cleaved caspase 3 antibody is validated for WB: https://www.cellsignal.com/products/primary-antibodies/cleaved-caspase-3-asp175-5a1e-rabbit-mab/9664
- rabbit anti-mouse Collagen I antibody is validated for IF: https://www.abcam.com/collagen-i-antibody-ab34710.html
- rabbit anti-mouse COX IV antibody is validated for WB: https://www.cellsignal.com/products/primary-antibodies/cox-iv-antibody/4844
- goat anti-mouse CRELD2 antibody is validated for WB: https://www.rndsystems.com/products/mouse-creld2-antibody_af3686
- rat anti-mouse F4/80 antibody is validated for IF: https://www.thermofisher.com/antibody/product/F4-80-Antibody-clone-BM8-Monoclonal/48-4801-82
- rabbit anti-mouse Fibronectin antibody is validated for WB & IF: https://www.abcam.com/fibronectin-antibody-ab2413.html
- rabbit anti-mouse FIS1 antibody is validated for WB: https://www.genetex.com/Product/Detail/FIS1-antibody/GTX111010
- rabbit anti-human & mouse Foxo3a antibody is validated for WB: https://www.cellsignal.com/products/primary-antibodies/foxo3a-d19a7-rabbit-mab/12829
- rabbit anti-human & mouse FUNDC1 antibody is validated for WB: https://www.cellsignal.com/products/primary-antibodies/fundc1-e2f4t-rabbit-mab/49240
- rabbit anti-human & mouse GAPDH antibody is validated for WB: https://www.cellsignal.com/products/primary-antibodies/gapdh-d16h11-xp-rabbit-mab/5174
- mouse anti-GFP antibody is validated for WB: https://www.sigmaaldrich.com/US/en/product/roche/11814460001
- rabbit anti-human & mouse IRF3 antibody is validated for WB: https://www.cellsignal.com/products/primary-antibodies/irf-3-d83b9-rabbit-mab/4302
- rabbit anti-human & mouse Phospho-IRF3 (Ser396) antibody is validated for WB: https://www.cellsignal.com/products/primary-antibodies/phospho-irf-3-ser396-d6o1m-rabbit-mab/29047
- rabbit anti-mouse Laminin antibody is validated for WB: https://www.abcam.com/laminin-antibody-ab11575.html
- rabbit anti-human & mouse LC3A/B antibody is validated for WB: https://www.cellsignal.com/products/primary-antibodies/lc3a-b-antibody/4108
- rabbit anti-human & mouse MANF antibody is validated for WB & IF: https://www.abnova.com/products/products_detail.asp?catalog_id=PAB13301
- goat polyclonal anti-human MANF antibody is validated for IF: https://www.rndsystems.com/products/human-manf-antibody_af3748
- rabbit anti-mouse Mitofusin2 antibody is validated for WB: https://www.cellsignal.com/products/primary-antibodies/mitofusin-2-d2d10-rabbit-mab/9482
- rabbit anti-human & mouse Phospho mTOR (Ser2481) antibody is validated for WB: https://www.cellsignal.com/products/primary-antibodies/phospho-mtor-ser2481-antibody/2974
- rabbit anti-human & mouse NF-kB antibody is validated for WB: https://www.abcam.com/nf-kb-p65-antibody-ab16502.html
- rabbit anti-human & mouse Phospho-NF-kB (Ser536) antibody is validated for WB: https://www.cellsignal.com/products/primary-antibodies/phospho-nf-kb-p65-ser536-93h1-rabbit-mab/3033

- goat anti-mouse NGAL antibody is validated for WB: https://www.rndsystems.com/products/mouse-lipocalin-2-ngal-antibody_af1857
- rabbit anti-mouse NKCC2 antibody is validated for IF: https://www.4adi.com/4adi/anti-rat-na-k-cl-cotransporters-2-nkcc2-bsc1-igg-1-aff-pure-12347-p.html
- mouse anti-rodent OXPHOS antibody is validated for WB: https://www.abcam.com/total-oxphos-rodent-wb-antibody-cocktail-ab110413.html
- rabbit anti-human & mouse P62/SQSTM1 antibody is validated for WB & IF: https://www.cellsignal.com/products/primary-antibodies/sqstm1-p62-antibody/5114
- mouse anti-human & mouse Parkin antibody is validated for WB: https://www.abcam.com/parkin-antibody-prk8-ab77924.html
- mouse anti-human & mouse PGC1a antibody is validated for WB: https://www.ptglab.com/products/PPARGC1A-Antibody-66369-1-Ig.htm
- rabbit anti-human & mouse PINK1 antibody is validated for WB: https://www.abcam.com/pink1-antibody-ab23707.html
- mouse anti-mouse Smooth muscle actin antibody is validated for WB & IF: https://www.sigmaaldrich.com/US/en/product/sigma/a2547
- rabbit anti-human STING antibody is validated for WB: https://www.cellsignal.com/products/primary-antibodies/sting-d2p2f-rabbit-mab/13647
- rabbit anti-mouse STING antibody is validated for WB: https://www.cellsignal.com/products/primary-antibodies/sting-d1v5l-rabbit-mab-rodent-preferred/50494
- rabbit anti-human & mouse TBK1/NAK antibody is validated for WB: https://www.cellsignal.com/products/primary-antibodies/tbk1-nak-d1b4-rabbit-mab/3504
- rabbit anti-human & mouse Phospho-TBK1/NAK (Ser172) antibody is validated for WB: https://www.cellsignal.com/products/primary-antibodies/phospho-tbk1-nak-ser172-d52c2-xp-rabbit-mab/5483
- rabbit anti-human & mouse TFAM antibody is validated for WB: https://www.sigmaaldrich.com/US/en/product/sigma/sab1401383
- rat anti-mouse Uromodulin antibody is validated for WB & IF: https://www.rndsystems.com/products/mouse-uromodulin-antibody-774056_mab5175
- rabbit anti-human & mouse Uromodulin antibody is validated for WB: https://alfaaesar.com:4433/en/catalog/J65429
- sheep anti-mouse Biotin labeled-Uromodulin antibody is validated for WB: https://www.rndsystems.com/products/mouse-uromodulin-biotinylated-antibody_baf5175
- mouse anti-human Uromodulin antibody is validated for WB & IF: https://www.raybiotech.com/mouse-anti-human-uromodulin/
- rabbit anti-human & mouse XBP1s antibody is validated for WB: https://www.biolegend.com/en-us/products/purified-anti-xbp-1s-antibody-6297

Eukaryotic cell lines

Policy information about cell lines and Sex and Gender in Research

Cell line source(s)	HEK-293 and 293T cells were purchased from ATCC, catalog number CRL-1573 and CRL-3216, respectively.
Authentication	Cell authentication is based on their morphology, growth conditions and specific gene expression.
Mycoplasma contamination	All cell lines used in this study were tested negative for mycoplasma contamination.
Commonly misidentified lines (See ICLAC register)	There were no commonly misidentified cell lines used in this study.

Animals and other research organisms

Policy information about studies involving animals; ARRIVE guidelines recommended for reporting animal research, and Sex and Gender in Research

Laboratory animals

Mice were maintained on a 12 hour light/dark cycle at 20-24 degree and controlled humidity (30-70%, usually around 50%) in an AAALAC accredited facility. Both male and female littermates ranging in age from 3-24 weeks were used for experiments.

- Umod DEL/+ mice were generated by Washington University Transgenic Vectors Core.
- TET-MANF mice were generated by Dr. Maria Lindahl.
- Umod C147W/+ mice on the C57BL/6 background were described and published previously (Johnson, B. G. et al. JCI 2017).
- Manf fl/fl on the C57BL/6 background was generated by Dr. Maria Lindahl and published previously (Lindahl, M. et al. Cell rep 2014).
- Umod IRES CRE-ERT2 on the C57BL/6 background was generated by Dr. Andrew P. McMahon in the University of South California and purchased from The Jackson Laboratory (Stock No: 030601).
- Pax8-rtTA mice on the C57BL/6 background were purchased from The Jackson Laboratory (Stock No: 007176). The hemizygous transgenic mice were intercrossed to generate the homozygous transgenic mice for some breedings by utilizing genomic DNA q-PCR genotyping.

Mice were observed closely for the signs of a premorbid state. These signs include hypoactivity; shallow, rapid, and/or labored breathing; failure to groom; failure to respond to stimuli; hunched posture; dehydration, and weight loss. Once these signs appeared, mice were euthanized. All surviving mice were euthanized at the end of experiment. Methods of euthanasia are consistent with the recommendations of the Panel on Euthanasia of the American Veterinary Medical Association. Mice were anaesthetized by intraperitoneal injection of ketamine/xylazine, followed by cervical dislocation.

Wild animals

The study does not involve wild animals.

Reporting on sex	Both sexes were used in the study. Sex was not tracked as a biological variable, as no difference related to the kidney disease phenotype was observed between the male and female littermates. The distribution of sex was equal across all groups.
Field-collected samples	The study does not involve samples collected from the fields.
Ethics oversight	Animal experiments conformed to the National Institutes of Health Guide for the Care and Use of Laboratory Animals were approved by the Washington University Animal Studies Committee (animal protocol number 20-0286).

Note that full information on the approval of the study protocol must also be provided in the manuscript.
